# Functional Dissection of the Bipartite Active Site of the Class I Coenzyme A (CoA)-Transferase Succinyl-CoA:Acetate CoA-Transferase

**DOI:** 10.3389/fchem.2016.00023

**Published:** 2016-05-23

**Authors:** Jesse R. Murphy, Elwood A. Mullins, T. Joseph Kappock

**Affiliations:** Department of Biochemistry, Purdue UniversityWest Lafayette, IN, USA

**Keywords:** 4FAC, 5DDK, 5DW4, 5DW5, 5DW6, 5E5H, substrate analog, enzyme mechanism, oxyanion hole, substrate-assisted catalysis

## Abstract

Coenzyme A (CoA)-transferases catalyze the reversible transfer of CoA from acyl-CoA thioesters to free carboxylates. Class I CoA-transferases produce acylglutamyl anhydride intermediates that undergo attack by CoA thiolate on either the internal or external carbonyl carbon atoms, forming distinct tetrahedral intermediates <3 Å apart. In this study, crystal structures of succinyl-CoA:acetate CoA-transferase (AarC) from *Acetobacter aceti* are used to examine how the Asn347 carboxamide stabilizes the internal oxyanion intermediate. A structure of the active mutant AarC-N347A bound to CoA revealed both solvent replacement of the missing contact and displacement of the adjacent Glu294, indicating that Asn347 both polarizes and orients the essential glutamate. AarC was crystallized with the nonhydrolyzable acetyl-CoA (AcCoA) analog dethiaacetyl-CoA (**1a**) in an attempt to trap a closed enzyme complex containing a stable analog of the external oxyanion intermediate. One active site contained an acetylglutamyl anhydride adduct and truncated **1a**, an unexpected result hinting at an unprecedented cleavage of the ketone moiety in **1a**. Solution studies confirmed that **1a** decomposition is accompanied by production of near-stoichiometric acetate, in a process that seems to depend on microbial contamination but not AarC. A crystal structure of AarC bound to the postulated **1a** truncation product (**2a**) showed complete closure of one active site per dimer but no acetylglutamyl anhydride, even with acetate added. These findings suggest that an activated acetyl donor forms during **1a** decomposition; a working hypothesis involving ketone oxidation is offered. The ability of **2a** to induce full active site closure furthermore suggests that it subverts a system used to impede inappropriate active site closure on unacylated CoA.

## Introduction

Substrate-dependent ordering of flexible active site loops can transmute substrate (ligand) binding affinity into faster enzymatic reactions (Jencks, 1975; Malabanan et al., 2010). When loop motions move critical functional groups, it is unclear if protein motions bias the enzyme-substrate complex conformational ensemble toward a reactive configuration or simply assemble the catalytic machinery. Enzymes with large substrates, like the coenzyme A (CoA)-based molecules used throughout metabolism, have ample opportunity to exploit substrate affinity to promote catalysis.

A classic example of this phenomenon is provided by the CoA-transferases, which activate metabolism of diverse carboxylate substrates by introducing the versatile and reactive CoA thioester (Moore and Jencks, 1982; Amyes and Richard, 1992; Yang and Drueckhammer, 2003), often at the expense of acetyl-CoA (AcCoA). *Acetobacter aceti* strain 1023 and other acetic acid bacteria use succinyl-CoA:acetate CoA-transferase (AarC) in a variant citric acid cycle that is required for a robust acetic acid resistance (*aar*) phenotype (Mullins et al., 2008). Strong selection for key roles in acetic acid resistance and central metabolism appear to have optimized the structural and functional properties of AarC, making it an excellent representative of the class I CoA-transferase superfamily.

Class I acyl-CoA:carboxylate CoA-transferases produce reactive acylglutamyl anhydride intermediates that acylate CoA, forming either an acyl-CoA product and a free enzyme or a protein glutamyl-CoA thioester and a carboxylate product. Jencks proposed that active site closure, which immobilizes the acyl thioester, potentiates catalysis (White and Jencks, 1976). Efficient catalysis requires an intact CoA, consistent with long-range mechanical coupling of remote “binding” regions to the site of chemistry (Fierke and Jencks, 1986; Whitty et al., 1995).

**Figure 1:**
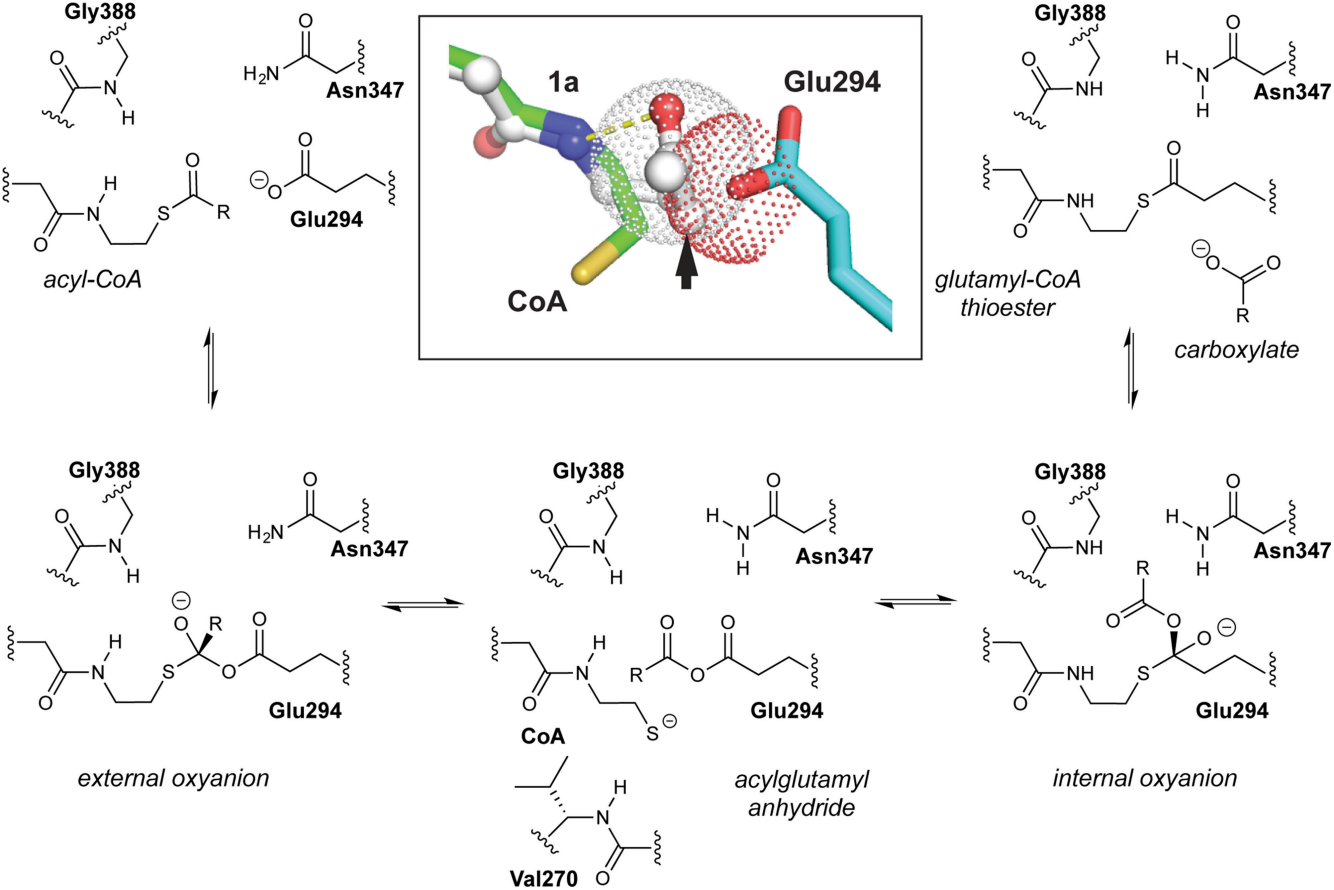
**Class I CoA-transferase mediated acyl transfers involve two spatially distinct oxyanion holes**. The reversible conversion of acyl-CoA substrate to the carboxylate product is shown, a half-reaction that converts free enzyme into a glutamyl-CoA thioester adduct. The other half-reaction reverses this reaction sequence. Inset, overlay of the “open” AarC•CoA complex (PDB entry 4eu7) with the “closed” AarC-E294A•**1a** complex (PDB entry 4euc) indicates that constriction of the active site around an acyl-CoA substrate causes extreme C/O overlap at an approach angle close to the ideal for nucleophilic attack on an unsaturated carbon (the Bürgi-Dunitz angle, 107°). The arrow indicates the position of the sulfur-for-methylene substitution in the AcCoA analog **1a**, which contains a nonhydrolyzable ketone.

The Jencks hypothesis was substantiated by a set of AarC crystal structures, including a trapped acetylglutamyl anhydride intermediate in which the CoA sulfur atom lies equidistant from two (external and internal) carbonyl carbon atoms (Mullins and Kappock, 2012). Competing thiolysis reactions (Figure 1) produce distinct tetrahedral oxyanion intermediates ~3 Å apart. The external oxyanion is stabilized by hydrogen bonds from CoA and Gly388 amides, whereas the internal oxyanion is stabilized by a hydrogen bond from the Asn347 carboxamide. While components of the external oxyanion hole are strictly conserved throughout the class I CoA-transferase superfamily, residues forming the internal oxyanion hole are supplied by different parts of the protein and are conserved within two large subsets of the superfamily.

**Figure 2:**
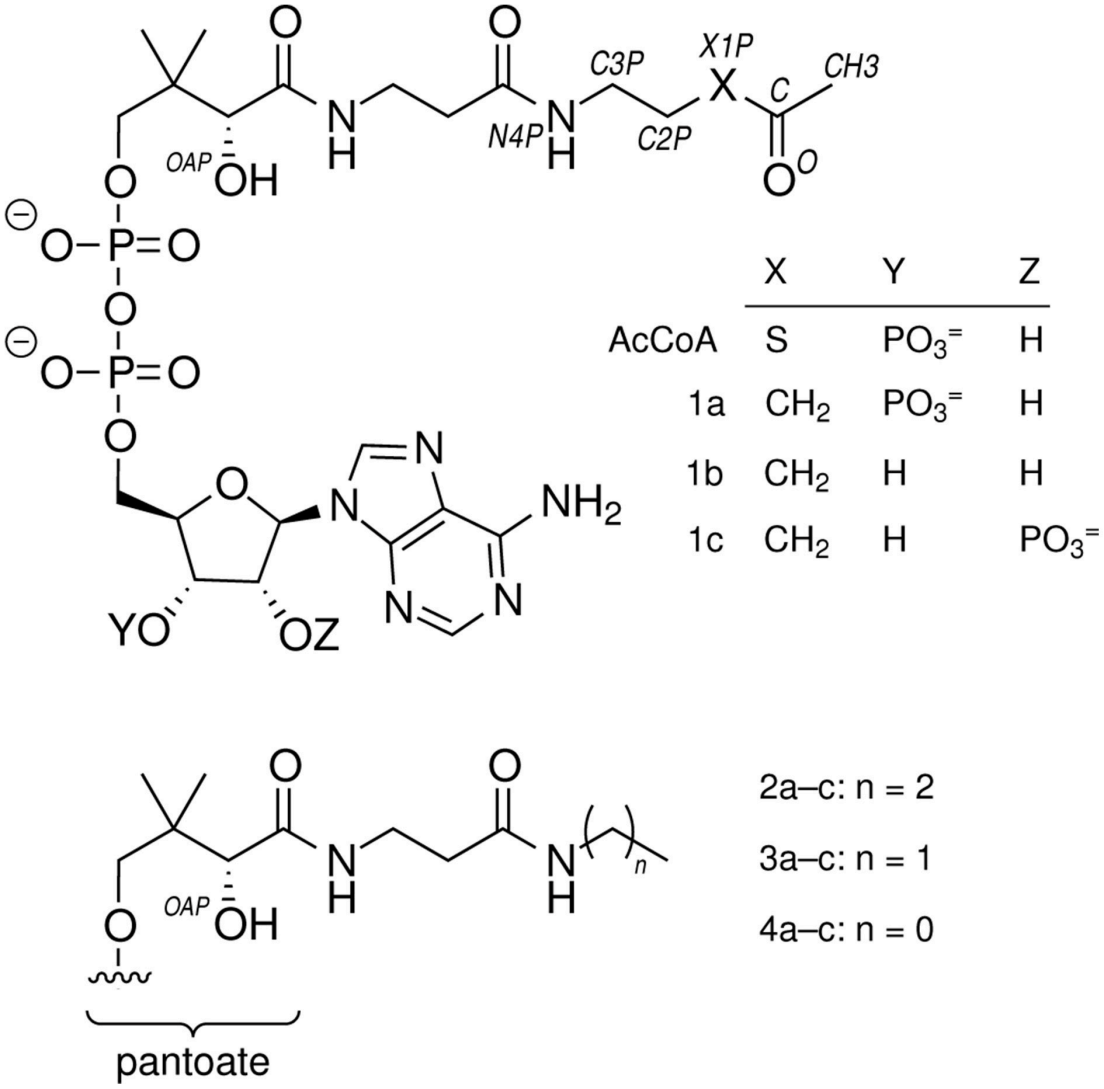
**Compounds referred to in this work**.

AarC crystal structures revealed that substrate binding provides the last piece of the external oxyanion hole, a hydrogen bond supplied by the CoA N4P hydrogen (Figure 1), and have begun to delineate steps in active site closure, a multi-step process that constricts the acyl-CoA substrate (Mullins and Kappock, 2012). Properly positioning Val270, a residue at the tip of one of two loops that move the most during active site closure, appears to be particularly important: its side chain is proposed to desolvate and constrain the thioester while its amino group is proposed to supply a hydrogen bond donor that stabilizes the CoA thiolate leaving group produced by anhydride formation. The amide-thiolate interaction would both stabilize the nucleophile that attacks the anhydride and help maintain the 270s loop in a closed state during reactions involving the anhydride adduct. A crystal structure of an inactive mutant, AarC-E294A, bound to the nonhydrolyzable AcCoA analog dethiaacetyl-CoA (Figure 2; **1a**), suggests that active site closure crushes the acyl-CoA thioester into the Glu294 nucleophile, enforcing a near-ideal Bürgi-Dunitz angle (Bürgi et al., 1973) and confining the thioester oxygen atom in the external oxyanion hole (Mullins and Kappock, 2012). In contrast, ligand binding seems to have little effect on the internal oxyanion hole, although the subsequent active site closure process may alter its dielectric environment.

In this study, we used crystal structures of active AarC forms to study enzyme closure and probe the assembly of both oxyanion holes. Mutagenesis of the internal oxyanion hole resulted in diminished but not completely lost enzyme activity (Mullins and Kappock, 2012). Since the external oxyanion hole is composed of backbone and CoA atoms, analogs incapable of undergoing complete enzymatic conversion were used to examine the assembly of a closed enzyme-ligand complex. Compound **1a** was unexpectedly degraded by microbial contamination, yielding a crystal containing an acetylglutamyl anhydride adduct and a CoA analog (modeled as **2a**). A complex of wild-type AarC and authentic **2a**, which deletes a hydrogen bond between the Glu294 carboxylate and the CoA thiol, showed complete closure of the active site. Since complete closure has not been observed in complexes of wild-type AarC and CoA, a specific polar contact formed between the Glu294 nucleophile and CoA may enable AarC to discriminate between acyl-CoA substrates and CoA. Selective prevention of complete active site closure on CoA might allow CoA-transferases to avoid forming dead-end complexes with ligands that cannot dissipate binding energy through enzyme catalysis.

## Experimental

### Materials and methods

#### Reagents and general analytical methods

Chemicals were purchased from Sigma-Aldrich (St. Louis, MO) or Fisher (Houston, TX) unless otherwise noted. Dimethylformamide (DMF) was dried over 3A molecular sieves under a N_2_ atmosphere. Oligodeoxynucleotides (ODNs) were obtained from Integrated DNA Technologies (Coralville, IA) and used without further purification. Yeast acetyl-CoA synthetase (catalog number A1765) and a rabbit pyruvate kinase/lactate dehydrogenase mixture (catalog number P0294) were from Sigma. DNA modifying enzymes were from New England Biolabs or Stratagene. Cells were disrupted at 4°C by three rounds of sonication (1 min on, 1 min cooling) using a Fisher Sonic Dismembrator 550. The synthesis of **1a** was described previously (Francois et al., 2006); no contaminating **1b** was detected (Figure 2). Absorbance measurements were recorded on a Cary Series UV-Vis spectrophotometer (Agilent; Santa Clara, CA) or Nanodrop 2000C (Thermo Scientific; Milwaukee, WI). Matrix assisted laser desorption ionization—time of flight mass spectrometry (MALDI-TOF MS) was performed on a 4800 Plus MALDI TOF/TOF (Farmington, MA). Daughter ion *m*/*z* values were computed assuming that adenine N1 can be protonated (Kapinos et al., 2011) or that the phosphates associate with either protons or potassium ions.

#### DNA manipulations

Plasmids pJK385, pJK513, and pJK524 encode AarC with a C-terminal hexahistidine tag (AarCH6), AarCH6-E294A, and AarCH6-N347A, respectively (Mullins and Kappock, 2012). For simplicity we will henceforth refer to AarCH6 proteins as AarC; the tagged and untagged proteins produce isostructural orthorhombic crystals (PDB entries 4eu7 and 4eud, respectively). Plasmids pESC124, pESC106, and pET15b/bPanK encode *Escherichia coli* CoaE, CoaD, and PanK proteins, respectively, each with an N-terminal hexahistidine tag (Calder et al., 1999; Strauss and Begley, 2002). Sequencing of pET15b/bPanK revealed a silent mutation (T → C) in the His104 codon that removes an internal, endogenous *Nde*I site. *E. coli* BL21(DE3) *ackA* was amplified using *Pfu* TURBO polymerase and oligodeoxynucleotides 2368 (5′-GCTGTCGCATATGTCGA GTAAGT) and 2369 (5′-ATT AGCTCGAGTCAGGCAGTC). The resulting PCR product was cloned into the *Nde*I and *Xho*I sites of pET28a to furnish plasmid pJK667, which is used to express AckA (acetate kinase) with an N-terminal hexahistidine fusion (H6AckA). Double-stranded DNA sequencing of all plasmids by the Purdue Genomics Core Low Throughput Laboratory yielded the anticipated sequence. All plasmids described in this section are available through Addgene (Herscovitch et al., 2012).

#### Production of CoA analogs 2a and 3a

CoA biosynthesis enzymes were overexpressed in *E. coli* BL21(DE3) cells transformed with pESC124, pESC106, or pET15b/bPanK and propagated on LB medium supplemented with 100 mg/L ampicillin or 70 mg/L kanamycin. Production cultures were grown with shaking at 220 rpm at 37°C to an optical density at 600 nm of 0.6, at which point isopropyl β-D-1-thiogalactopyranoside was added (0.4 mM final concentration). After an additional 4 h at 37°C, cells were harvested by slow centrifugation (5000 g, 15 min) and stored at −80°C.

H6AckA was produced in *E. coli* BL21(DE3) cells transformed with pJK667 and propagated on LB medium supplemented with 100 mg/L ampicillin. Production cultures of ZYM-5052 autoinduction medium (1 L) (Studier, 2005) were grown with shaking at 220 rpm at 37°C for 18 h. Cells were harvested by slow centrifugation and stored at −80°C.

The following protocol was used to isolate H6AckA, H6PanK, H6CoaD, or H6CoaE; all steps were performed at 4°C. Cells were resuspended in 5 mL/g TM buffer (50 mM Tris-HCl, pH 8.0) at 4°C and disrupted by sonication (3 rounds of 1 min on and 1 min off, 20% intensity). Debris was removed by fast centrifugation (30,000 g, 30 min). The supernatant was adjusted to 1% (w/v) streptomycin sulfate [10% (w/v) stock] and solids were removed by fast centrifugation. The supernatant was applied under gravity flow to a Ni^2+^—iminodiacetic acid Sepharose column (2.5 × 8 cm, ~5 mL) in TM buffer. After washing with TM buffer containing 300 mM KCl and 40 mM imidazole (50 mL), bound proteins were displaced using TM buffer containing 300 mM KCl and 500 mM imidazole (20 mL). SDS-PAGE was used to identify fractions containing the relevant proteins. Fractions were pooled, dialyzed 18 h against 50 mM Tris-HCl, pH 8.0, 100 mM KCl (1 L × 2 changes), and either adjusted to 50% (v/v) glycerol and stored at −20°C or flash-frozen (H6AckA) in liquid N_2_ and stored at −80°C. Specific activities were determined for H6AckA (Ferry, 2011) and H6PanK (Francois et al., 2006).

*N*-propylpantothenamide and *N*-ethylpantothenamide were synthesized as described previously (Strauss and Begley, 2002). Sodium pantothenate (2.0 g, 8.3 mmol) was converted to the free acid using a Dowex 50W (H^+^ form) column (1.7 × 10 cm). Pantothenic acid (1.7 g, 7.8 mmol) was dissolved in 10 mL dry DMF and either propylamine or ethylamine (0.82 mL, 10 mmol) was added dropwise with continuous stirring under a N_2_ atmosphere at 22°C. Diphenylphosphoryl azide (2.2 mL, 10 mmol) was then added dropwise and the reaction mixture was placed in an ice bath. After 10 min, triethylamine (1.39 mL, 10 mmol) was added dropwise and the reaction mixture was stirred at 0°C for 2 h, then 22°C for 15 h. A portion (0.3 mL) of the reaction mixture above was mixed with deionized water (1.2 mL), solids were removed by centrifugation (16,000 g, 10 min), and solvent was removed under reduced pressure at 60°C. The resulting clear, viscous oil was dissolved to give a ~0.1 M aqueous solution. A final volume of 5 mL contained 50 mM Tris-HCl, pH 8.0, 5 mM MgCl_2_, 5 mM ATP, 300 μg H6PanK (2.7 units), 300 μg H6CoaD, 1500 μg H6CoaE, and either 10 mM *N*-propylpantothenamide or *N*-ethylpantothenamide. After 4 h at 37°C, 0.5 mL formic acid (98%) was added and solids were removed by centrifugation (16,000 g, 10 min). The quenched reaction mixture was injected (5 mL) onto an Agilent 110 series HPLC equipped with a Luna 5 μm C18(2) 250 × 21.2 mm column equilibrated in 0.1% (v/v) trifluoroacetic acid (TFA) and 2% methanol. The column was developed in a gradient of 2–90% methanol in 0.1% TFA and large peaks were collected, frozen, and lyophilized to dryness. The resulting solid, containing **2a** or **3a** (Figure 2), was resuspended in 5 mM HCl and concentrations were determined by the absorbance at 260 nm assuming a molar extinction coefficient of 16.4 mM^−1^cm^−1^. The molecular mass and purity of 3′-phosphoadenosine 5′-(*O*-(*N*-propyl-*R*-pantothenamide))pyrophosphate (**2a**) and 3′-phosphoadenosine 5′-(*O*-(*N*-ethyl-*R*-pantothenamide))pyrophosphate (**3a**) were assessed using LCMS (Agilent 1100 HPLC G1946B).

#### AarC production and characterization

AarC and AarC mutants were expressed in C41(DE3) pREP4-*groESL*, purified (Figure S1), and characterized by VisR and LCR activity assays as described previously (Mullins et al., 2008; Mullins and Kappock, 2012). Sodium borohydride inactivation experiments were performed as described previously (Mullins et al., 2008). Activities for AarC and other enzymes are expressed in units, defined as 1 μmol product formed per min.

#### Crystal growth and X-ray data collection

Crystals were grown at 22°C using the hanging-drop vapor-diffusion method by modifying a published method (Mullins and Kappock, 2012). Reservoir solutions (0.5 mL) contained 0.9 M sodium citrate, 0.1 M imidazole-HCl, pH 8.2, and 25 mM 2-mercaptoethanol. Drops contained 2 μL of reservoir solution mixed with 2 μL of protein solution (6.0 mg/mL AarC, 45 mM Tris-HCl, pH 8.0, 90 mM KCl, either 10 mM CoA, 10 mM **1a**, or 1–3 mM **2a**). Some drops contained 1–50 mM sodium acetate, pH 8.2, instead of (or in addition to) a CoA analog. Three days prior to cryoprotection, sodium acetate (~50 mM final, added from a 1 M stock at pH 8.2) was gently added to several drops containing crystals grown in the presence of **2a**. Crystals were soaked for 13 h in a cryoprotectant solution containing 15% (w/v) sorbitol, 1.1 M sodium citrate, 0.1 M imidazole-HCl, pH 8.2, 25 mM 2-mercaptoethanol, and any additional ligands (each at 110% of the concentration used for crystallization). No special measures were undertaken to exclude microbial contaminants. Crystals were loaded into Nylon loops, flash-cooled by rapid immersion in liquid N_2_, and kept at or below 100 K (Teng, 1990). X-ray diffraction data were collected at LS-CAT beamlines at the Advanced Photon Source at Argonne National Laboratory. Diffraction data were indexed, integrated, and scaled using HKL2000 (Otwinowski and Minor, 1997).

#### Determination, refinement, and analysis of crystal structures

Automatic and manual refinement were performed using PHENIX (Adams et al., 2010) and Coot (Emsley et al., 2010), respectively. Ligand coordinates and restraints were obtained from HIC-Up (Kleywegt, 2007) and modified using PHENIX.

All structures were solved using a hybrid model of translation-libration-screw (TLS) groups and isotropic atomic *B*-factor (*B*_iso_) terms for all protein atoms. PHENIX analysis of the 4eu9 coordinates was used to define a set of 12 TLS groups. The starting model for direct refinement was protein atoms from AarC-R228E•CoA (PDB entry 4eu9) coordinates, with initial *B*_iso_ values set to 30 Å^2^ and minor alternative conformations set to zero occupancy. Initial TLS refinement (15 cycles) (Merritt, 2012) was followed by positional (Cartesian, real-space, and rigid-body) and ADP refinement (5 cycles). CoA or an analog was then added (except PDB entry 5dw4) and superfluous alternate conformations were deleted using COOT. Subsequent refinement rounds omitted rigid-body refinement, added occupancy refinement, and were alternated with manual model adjustments in COOT. Ligands, known buffer components, and hypothetical **1a** degradation products (smaller CoA analogs and, in one structure, a formate molecule) were built into continuous difference electron density. Cys15 in some structures showed evidence of sulfur oxidation and was modeled as cysteine sulfenic acid (CSX form; Furdui and Poole, 2014). Riding hydrogens used during positional refinement steps were deleted before a final round of ADP refinement (10 cycles).

Structures were checked using MOLPROBITY (Chen et al., 2010) and the worldwide Protein Data Bank (Berman et al., 2003) validation pipeline (Read et al., 2011). Cavities and pores were identified using MOLEonline 2.0 (Sehnal et al., 2013). Several figures were prepared using Pymol (DeLano, 2002).

#### Stability of 1a and 2a

A final volume of either 0.5 or 1.0 mL containing 50 mM potassium phosphate, pH 8.0, 100 mM KCl, 100 μM **1a**, and 10 μM (subunit concentration) of either AarC (21.5 units, 0.28 mg or 43 units, 0.56 mg) or AarC-E294A (≤0.0043 units, 0.56 mg) was incubated at 22°C in a polypropylene microcentrifuge tube. One complete AarC reaction mixture was sterile-filtered (surfactant-free cellulose acetate syringe filter with 0.22 μm pores; Nalgene, Rochester, NY) to remove microbes and placed in a sterile, silanized microcentrifuge tube (MidSci; St Louis, MO). A no-enzyme control and reaction mixtures containing 10 μM **2a** instead of **1a** were processed in parallel. Aliquots (0.1 mL) were withdrawn periodically, placed in a microcentrifuge tube, and heated in a sand bath (95°C, 15 min). Solids were removed by centrifugation (16,000 g, 10 min), an ultraviolet spectrum was recorded, and the clarified reaction mixture was stored at −20°C. **2a** derivatives were quantitated using a Waters (Milford, MA) Breeze HPLC system equipped with an Agilent (Santa Clara, CA) Zorbax Eclipse XBD-C18 column (4.6 × 150 mm, 5 μm) and a Waters 717 autosampler. A portion of each quenched reaction mixture (20 μL) was mixed with an equal volume of 10% (w/v) trichloroacetic acid (TCA) and applied to the column, which was developed isocratically [200 mM sodium phosphate, 150 mM sodium acetate, pH 4.6, adjusted to 2% (v/v) acetonitrile] at 26°C at a flow rate of 1 mL/min with detection at 260 nm. CoA analogs were identified by comparison to standard mixtures (run daily to correct for variable retention patterns) and their concentrations were determined by reference to the analog peak area in an immediately quenched (*t* = 0) complete reaction mixture.

Additional small aliquots (2.7 μL) were periodically removed from reaction mixtures, either containing or lacking **1a**, and diluted to a final volume of 0.15 mL in phosphate/KCl buffer with or without 10 mM sodium borohydride. After 10 min at 25°C, aliquots (5 μL) were removed and residual SCACT activity was measured in LCR assays (Mullins et al., 2008).

#### Identification of acetate

A 1 M, pH 8.0 acetate standard was prepared from solid sodium acetate. A final volume of 20 μL contained 50 mM potassium phosphate, pH 8.0, 100 mM KCl, 25 mM MgCl_2_, 16 μL of either the quenched **1a** degradation mixture or an acetate standard, 1.0 mM CoA, 1.8 mM ATP, and acetyl-CoA synthetase (0.004 units) at 22°C. After 45 min, TCA [20 μL, 10% (w/v)] was added and solids were removed by centrifugation (16,000 g, 10 min). Waters HPLC analysis (25 μL injections) revealed a single large peak, not present in a control reaction mixture lacking acetyl-CoA synthetase, that co-migrated with an authentic AcCoA standard. AcCoA concentrations, determined by reference to AcCoA standard injections, were taken to be the lower limit for acetate production in **1a** stability assays.

#### Quantitation of acetate

A final volume of 70 μL in a microcentrifuge tube contained 50 mM potassium phosphate, pH 8.0, 100 mM KCl, 6.7 mM MgCl_2_, 10 μL of either a quenched **1a** stability assay reaction mixture or an acetate standard, 0.14 mM NADH, 1 mM ATP, 2 mM phosphoenolpyruvate (PEP), H6AckA (3 units), pyruvate kinase (6 units), and lactate dehydrogenase (6 units) at 22°C. Reactions were initiated by the addition of H6AckA. After 40 min, the entire reaction mixture was transferred to a black-walled microvolume quartz cuvette (70 μL capacity) and a spectrum was recorded. Acetate concentrations were determined by subtracting the absorbance at 340 nm (A_340_) of a control reaction lacking H6AckA (Δε_340_ = 6.22 mM^−1^ cm^−1^).

#### MALDI-TOF analysis of 1a degradation products

Quenched **1a** stability assay reaction mixtures were thawed and an aliquot (1 μL) was mixed with 9 μL 1% (v/v) formic acid. A portion of the acidified mixture (0.5 μL) was mixed with 0.5 μL matrix solution (1 mg/mL α-cyano-4-hydroxycinnamic acid in 50% (v/v) acetonitrile and 0.1% (v/v) TFA), deposited on a stainless steel MALDI plate, and air-dried for ~2 h. Analysis was performed in positive ion mode. Batch method cation exchange (PSE cation exchange resin) and solid phase extraction using ZipTip pipette tips (Millipore) was also attempted on **1a** stability assay time points.

## Results

### Parameterized active site conformations

**Figure 3:**
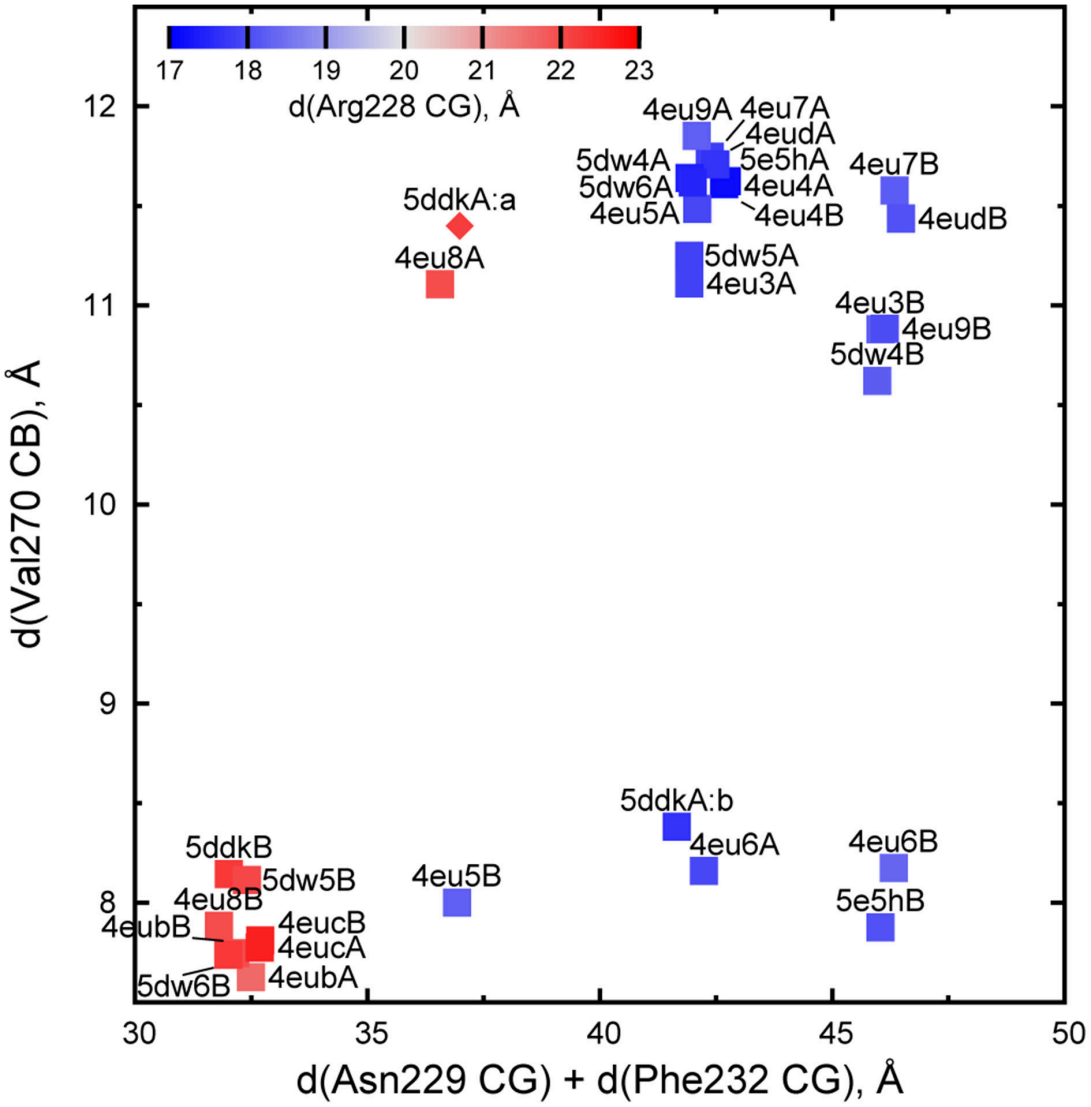
**Parametrization of AarC(H6) active site conformations**. Each point represents one subunit of AarC (PDB entry 4eud) or AarCH6 and its mutant forms (remaining points). The values are derived from distances (d) between Gly388 N, a fixed reference point within the active site, and the indicated atom(s) in a wild-type or mutant subunit. All proteins crystallized in an orthorhombic lattice apart from 4eu4, which is in a hexagonal lattice (Mullins and Kappock, 2012). Note that the two hexagonal subunits adopt almost identical open conformations. The open conformations of subunit B in orthorhombic crystals form a sub-cluster (upper right) due to crystal contacts described in the text. One Val270 CB position was estimated for one minor conformer (5ddkA:a, diamond symbol) using real-space refinement.

A closed active site enables AarC to engage, immobilize, and desolvate acyl-CoA substrates and to stabilize covalent adducts of Glu294. Crystal structures revealed four stages in active site closure, ranging from fully open (exemplified by AarC, PDB entry 4eu3) to fully closed (exemplified by AarC-E294A•CoA, PDB entry 4eub), each associated with characteristic 230s loop (residues 228–234) and 270s loop (residues 270–274) conformations (Mullins and Kappock, 2012). Given the underlying complexity of active site loop dynamics, we sought to replace discrete stages with continuous measures that allow objective structural comparisons. The comparatively immobile external oxyanion hole component Gly388 N was chosen to be the reference point for four interatomic distances that capture 230s loop motion (the sum of distances to Asn229 CG and Phe232 CG), active site constriction (the distance to Val270 CB), and the in/out conformations of Arg228 (the distance to Arg228 CG), which occur twice per half-reaction and are typically reciprocal to the movements of Asn229. This parametrization groups closed conformations (cluster at lower left in Figure 3) and open conformations (upper right in Figure 3) and allows ready identification of intermediate states such as those associated with covalent enzyme adducts (e.g., the acetylglutamyl anhydride 4eu6A and the glutamyl-CoA thioester 4eu6B).

Different sub-clusters for subunit A and B open conformations (Figure 3) arise from crystal-packing interactions in the orthorhombic lattice adopted in the majority of AarC(H6) crystals. The loop containing Phe232B contacts helix α4 in subunit A from a neighboring asymmetric unit, which leads to a slightly larger separation of this region from a reference point in each active site [the intra-subunit Phe232 CG-Gly388 N distance is 21.7 ± 0.1 Å (*n* = 9) in subunit A and 24.9 ± 0.1 Å (*n* = 5) in subunit B]. An open-conformation structure solved in a hexagonal lattice (PDB entry 4eu4) has no comparable crystal-packing contacts and resembles orthorhombic subunit A (the Phe232 CG-Gly388 N distance is 21.9 Å for both subunits). The other three residues considered appear to be unaffected by subunit-specific differences in crystal packing.

### A misaligned nucleophile in AarC-N347A

AarC-N347A lacks the carboxamide moiety proposed to contact the internal oxyanion formed during thiolysis of the acylglutamyl anhydride intermediate (Figure 1). The mutant retains appreciable (~15% of wild-type) specific activity (Mullins and Kappock, 2012). A data set obtained from a crystal grown in the presence of CoA (Table 1) was used to determine a structure in an orthorhombic space group (Table 2). Like all structures described here, the protein fold and CoA binding site were the same as described previously (Mullins and Kappock, 2012). Chloride ions were bound, as expected, near the pseudo-twofold axis or between C-terminal domains of each subunit. Three imidazole ligands were also included at the latter interface.

**Table 1:** **Crystallographic data collection statistics^a^**.

**PDB ID**	**5ddk**	**5dw4**	**5dw5**	**5dw6**	**5e5h**
Enzyme form	N347A	wt	wt	wt	wt
Ligand(s) added	CoA	acetate	**2a**	acetate, **2a**	**1a**
Diffraction source	21-ID-G	21-ID-F	21-ID-F	21-ID-F	21-ID-F
Wavelength (Å)	0.97856	0.97872	0.97872	0.97872	0.97872
Space group	*P*2_1_2_1_2_1_	*P*2_1_2_1_2_1_	*P*2_1_2_1_2_1_	*P*2_1_2_1_2_1_	*P*2_1_2_1_2_1_
*a, b, c* (Å)	67.103, 109.775, 120.057	67.503, 110.393, 120.021	67.477, 110.305, 120.144	67.425, 110.455, 120.003	67.192, 109.844, 119.682
α, β, γ (°)	90.0, 90.0, 90.0	90.0, 90.0, 90.0	90.0, 90.0, 90.0	90.0, 90.0, 90.0	90.0, 90.0, 90.0
Resolution range (Å)	50–2.13 (2.17–2.13)	50–1.62 (1.65–1.62)	50–1.66 (1.69–1.66)	50–1.55 (1.58–1.55)	50–2.05 (2.09–2.05)
No. of reflections	680,538	957,669	819,908	1,051,037	731,083
No. of unique reflections	50,048 (2441)	113,965 (5650)	106,546 (4370)	130,308 (6457)	55,051 (2724)
Completeness (%)	99.1 (98.0)	100 (100)	99.0 (82.4)	100 (100)	98.0 (97.3)
Multiplicity	13.6 (13.1)	8.4 (7.7)	7.7 (7.1)	8.1 (7.8)	13.3 (12.7)
I/σ(I)	22.9 (4.1)	30.47 (4.02)	50.19 (6.10)	37.4 (3.32)	24.4 (4.4)
*R*_sym_	0.116 (0.652)	0.096 (0.573)	0.059 (0.307)	0.075 (0.601)	0.125 (0.640)
Wilson *B*-factor (Å^2^)	20.1	11.9	19.6	14.8	18.4

a Values given in parentheses are for the highest-resolution shell.

**Table 2:** **Crystallographic refinement statistics^a^**.

**PDB ID**	**5ddk**	**5dw4**	**5dw5**	**5dw6**	**5e5h^b^**
Resolution range (Å)	32.34–2.13 (2.17–2.13)	44.85–1.62 (1.64–1.62)	44.87–1.66 (1.68–1.66)	44.82–1.55 (1.57–1.55)	41.39–2.05 (2.09–2.05)
Completeness (%)	95 (85)	100 (95)	99 (86)	100 (87)	98 (96)
No. of reflections, working set	47,881 (2395)	113,873 (3410)	106,455 (2885)	130,205 (3586)	54,907 (2552)
No. of reflections, test set	2394 (119)	5691 (175)	5344 (157)	6492 (189)	2740 (132)
*R*_work_	0.1449 (0.1706)	0.1502 (0.1959)	0.1504 (0.1743)	0.1566 (0.2100)	0.1511 (0.1802)
*R*_free_	0.1920 (0.2323)	0.1767 (0.2492)	0.1737 (0.2076)	0.1787 (0.2372)	0.1937 (0.2379)
No. of non-H atoms					
Protein (all atoms)	7815	7890	7841	7867	7827
Ligand	121	49	122	135	118
Water	623	1017	885	939	598
Total	8559	8956	8848	8941	8543
R.m.s. deviations					
Bonds (Å)	0.004	0.007	0.007	0.009	0.014
Angles (°)	0.859	1.136	1.124	1.231	1.369
Average *B*-factors (Å^2^)					
Protein (all atoms)	26.5	16.7	25.9	21.6	24.3
Ligand	25.9	29.9	29.8	24.6	34.7
Water	31.6	28.7	36.2	32.5	29.2
Total	26.8	18.2	27.0	22.8	24.8
Ramachandran plot					
Favored regions (%)	96.2	97.3	97.0	96.9	97.1
Additionally allowed (%)	3.7	2.5	2.9	3.1	2.8
Outliers (%)	0.1	0.2	0.1	0.0	0.1
Clash score^c^^,^^d^	2.92 [99]	4.09 [97]	3.59 [98]	3.00 [98]	2.15 [99]
MolProbity overall score^d^	1.34 [99]	1.32 [96]	1.32 [97]	1.27 [96]	1.15 [100]

a Values given in parentheses are for the highest-resolution shell.

b The B_iso_-only refinement strategy described previously (Mullins and Kappock, 2012) was used to obtain a model based on the same data set (PDB entry 4fac). Since the 4fac and 5e5h models are very similar, only the latter will be considered.

c Clash score is the number of overlaps >0.4 Å per 1000 atoms (Chen et al., 2010).

d Percentile scores, given in brackets, are relative to crystal structures determined at comparable resolution (Chen et al., 2010).

**Figure 4:**
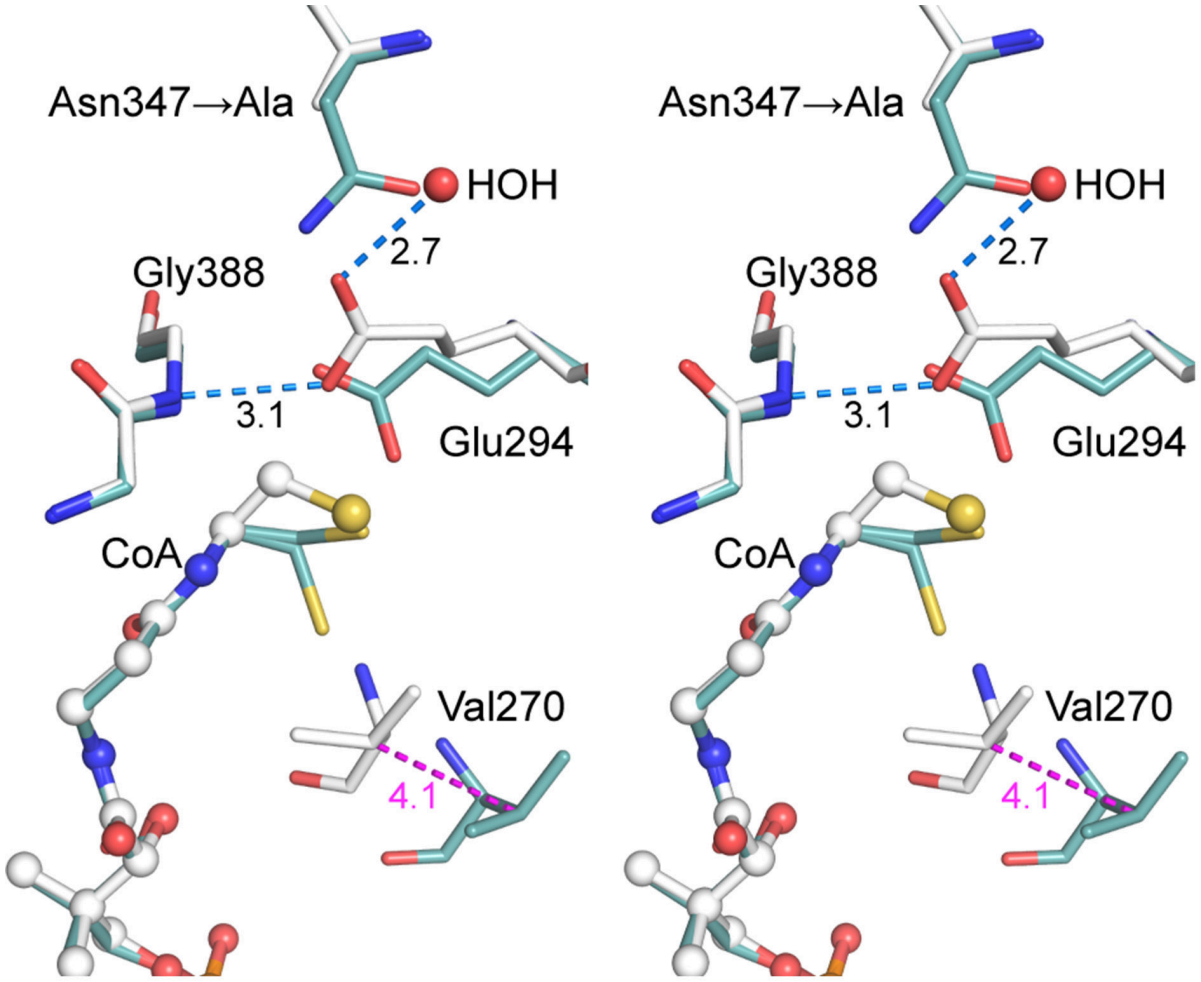
**Stereogram of the AarC-N347A active site**. The B subunits of AarC-N347A (PDB entry 5ddk, white carbon atoms) and AarC (PDB entry 4eu7, blue carbon atoms), each bound to CoA, are superposed. Spheres are shown for ligand atoms in AarC-N347A. The Glu294 carboxylate shifts by about the width of the carboxylate moiety, allowing a different oxygen atom to interact with Gly388 N (separated by 0.33 Å here). The Glu294 rotamer changes from *mt-10* (open) to *tt-0* (closed), as illustrated here, but the closed χ_3_ value is unusual. Distances (Å) in blue show hydrogen bonds involving the Glu294 nucleophile, including one to the “new” HOH in the AarC-N347A•CoA complex. The distance colored magenta depicts the motion of Val270 CB between open (AarC) and closed (AarC-N347A) conformations.

The final refined model (PDB entry 5ddk) contained alternate conformations for both CoA ethanethiol moieties and residues Arg228A-Asp236A, with the major and minor contributions from closed and open conformations, respectively. The subunit A 230s loop conformations differ by tandem flips of the two peptide bonds to Asn229. The subunit A 270s loop adopted multiple conformations, with the closed conformation most abundant. Difference electron density peaks consistent with the 270s loop open conformation were observed but too weak to justify inclusion in the final model. All three regions in subunit B adopted the closed conformation. The differences in CoA complex structures for wild-type and mutant AarC (open and closed, respectively; subunit B) do not affect this region of the active site (Figure 4).

Since AarC-N347A retains significant activity, our hypothesis was that a polar group would simply supplant the missing Asn347 carboxamide and restore its function. As anticipated, an active site solvent molecule was observed at this location in each subunit, with a lower *B*-factor in subunit B (HOH 775A and 787B: 58 and 34 Å^2^, respectively). The solvent substitution, however, additionally appears to influence the conformation of Glu294. While Glu294 in the N347A mutant adopts the tt-0 rotamer observed in other closed structures, its χ_3_ value is unusual, corresponding to a rotation of the attacking oxygen toward the mutated residue (i.e., away from the CoA binding site) [Any value of χ_3_ is permitted in the Glu tt-0 rotamer (Lovell et al., 2000)]. The resulting misalignment of the key carboxylate may decrease the likelihood of productive nucleophilic attack on an acyl-CoA substrate and affect the reactivity of covalent glutamyl adducts.

### Conversion of 1a during crystallization with AarC

**Figure 5:**
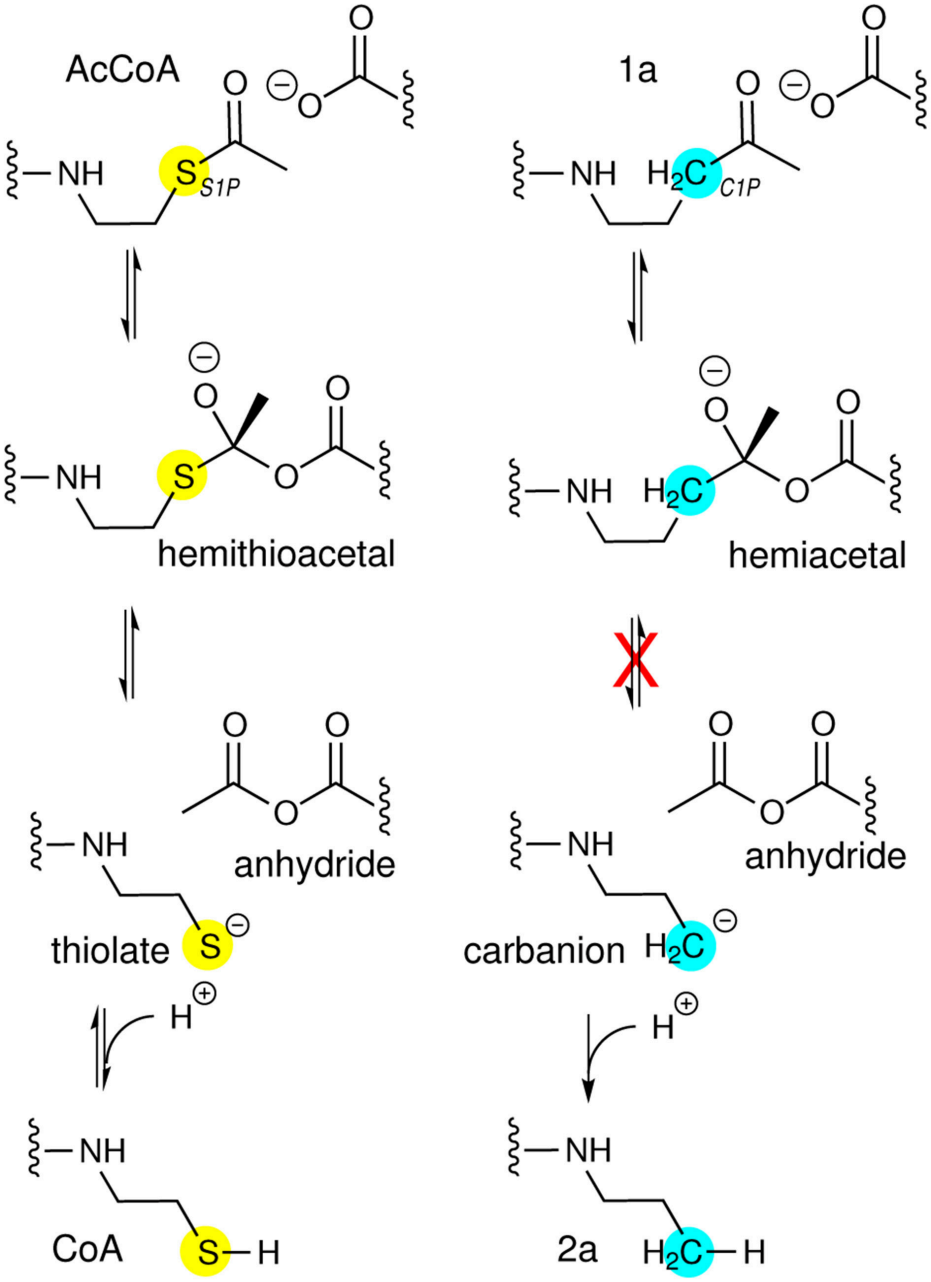
**CoA analogs as probes of CoA-transferase mechanism**. **(Left)** Reactions involving the external carbonyl of the acetylglutamyl anhydride. Thiolate protonation is possible but unlikely to be part of the normal reaction coordinate. **(Right)** Attempted trapping of a tetrahedral external oxyanion intermediate. The atomic substitution in **1a** has comparatively small effects on electronegativity and sterics so could allow formation of a hemiacetal intermediate resembling the external oxyanion and perhaps enable insight into how its reactivity is modulated. The tetrahedral intermediate formed by nucleophilic attack by AarC on the **1a** carbonyl cannot be resolved into cleavage products. The very high pK_a_ of an unactivated alkyl methyl, perhaps 50, would preclude formation of the alkyl carbanion required, even considering the exothermic subsequent protonation.

The AcCoA ketone analog **1a**, in which the thioester sulfur atom is replaced by a methylene group, was tested for its ability to form a hemiacetal analog of the tetrahedral hemithioacetal intermediate stabilized by the external oxyanion hole (Figure 5). Cleavage of a **1a**-derived hemiacetal would be prevented by the extremely poor leaving group potential of an alkyl carbanion, in principle yielding a stabilized external oxyanion. AarC-E294A readily formed crystals containing **1a** in a closed active site (PDB entry 4euc). Structure comparisons led us to predict that a severe steric clash with the Glu294 carboxylate (Figure 1, insert) would favor anhydride formation but might preclude formation of a closed AarC•**1a** complex (Mullins and Kappock, 2012).

**Figure 6:**
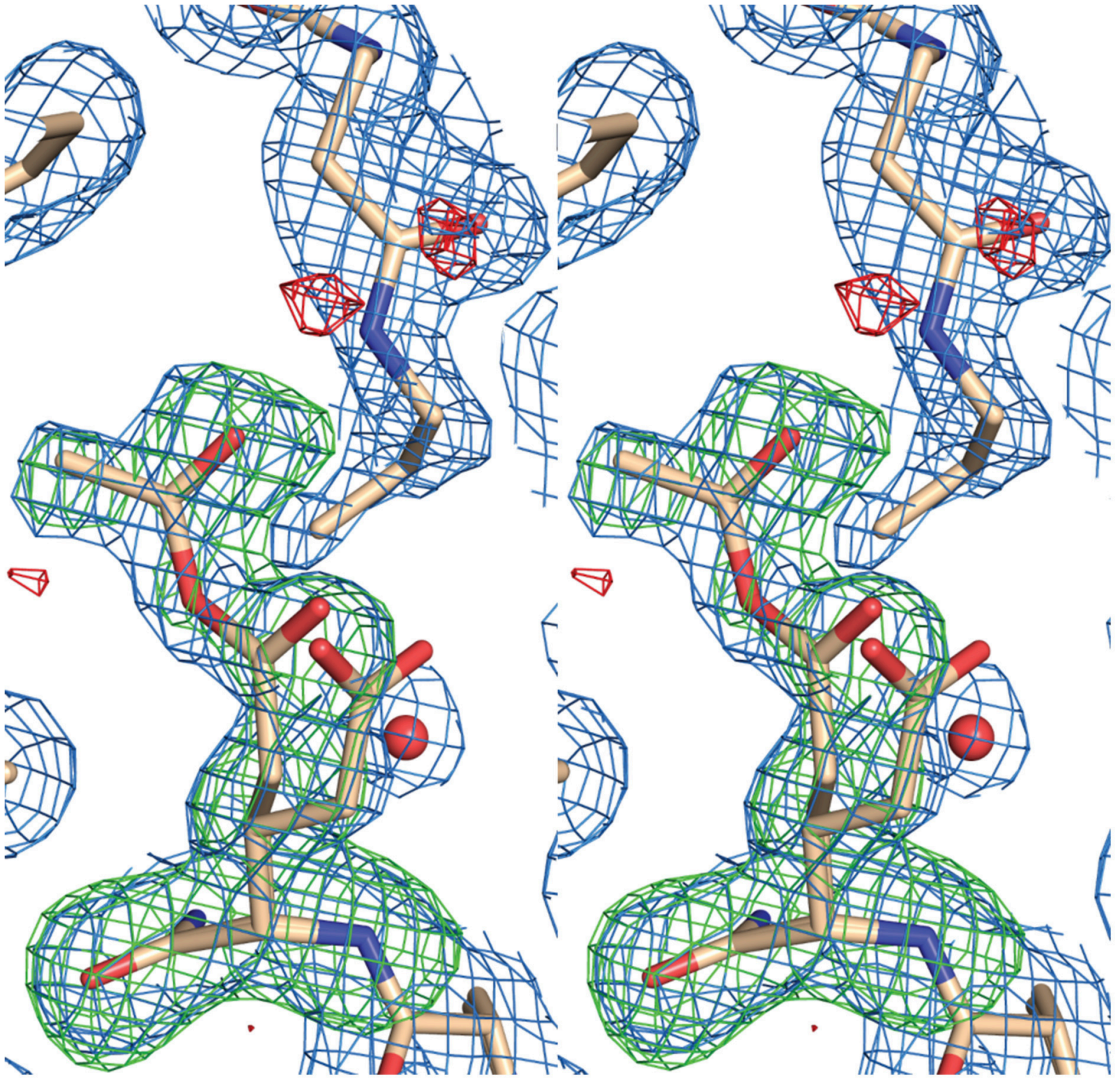
**Stereogram of electron density in the active site for AarC crystals grown in the presence of 1a**. Glu294A, located below the **1a**-derived compound modeled as **2a**, is present as a mixture of 39% free carboxylate and 61% acetylglutamyl anhydride. Atoms in Glu294A and the acetyl group (ACE 604A) were removed from the model prior to computing an omit map. Electron density maps are shown at 0.8 σ with a 2 Å carve radius (2mFo-DFc, blue mesh), −3 σ (mFo-DFc omit, red mesh), or +3 σ (mFo-DFc omit, green mesh).

AarC was crystallized in the presence of **1a** and its structure was determined (Table 2; PDB entry 5e5h). Despite our expectations, electron density for Glu294A was most consistent with a mixture composed of both free carboxylate and acetylglutamyl anhydride adduct in a 0.4/0.6 ratio, with a relatively narrow range of *B*-factors (Figure 6). Since density associated with the 3′-phosphate was ambiguous and weak, the large ligand in subunit A was modeled as **2b** (Figure 2). Excess density was noted near the 2′-hydroxyl that might be due to a minor contribution from a 2′-phospho isomer (**2c**). [Iso-CoA forms during the chemical synthesis of CoA (Burns et al., 2005).] The electron density associated with the large ligand in subunit B did not extend beyond carbon atom C3P, so it was modeled as 3′-phosphoadenosine 5′-(*O*-(*N*-methyl-*R*-pantothenamide))pyrophosphate (Figure 2; **4a**). Unlike subunit A, convincing electron density was associated with the 3′-phosphate group in subunit B, stabilized by the side chain of Lys408B. An active site acetate ligand was also included in subunit B.

Subunit A (5e5hA) adopted an open conformation distinctly different from a partially closed conformation observed for an AcCoA-derived acetylglutamyl anhydride (PDB entry 4eu6A) (Figure 3). The former appears to be less able to shield a labile acetylglutamyl anhydride from hydrolysis. Subunit B (5e5hB) adopted a partially closed conformation similar to that observed for the glutamyl-CoA thioester adduct bound to acetate (PDB entry 4eu6B) (Figure 3), even though **1a** and truncated derivatives (e.g., **2a** and **4a**) cannot form thioesters.

Acetate observed previously in an AcCoA-soaked AarC crystal, in an active site containing a glutamyl-CoA thioester (PDB entry 4eu6B), was presumed to arise from AcCoA hydrolysis (Mullins and Kappock, 2012). No chemical process of which we are aware could convert the ketone in **1a** to **2a** and acetate, let alone an acetylated molecule capable of producing an anhydride. The apparent formation of an anhydride seems to imply that AarC converts the **1a** ketone into a two-carbon activated acetyl group, with the remaining atoms forming **2a** or a related compound. No CoA-transferase has been reported to perform oxidation activity, suggesting that the conversion of **1a** to an AarC acylating agent may be due to an unknown contaminating enzyme or enzymes. Subsequent hydrolysis (n.b., not the normal thiolysis) of the anhydride would account for the formation of acetate in subunit B. Positive evidence for these transformations cannot be gleaned from electron density maps alone. We therefore attempted to obtain chemical evidence for the formation of **1a**-derived compounds. Partial disorder of the **1a** ketone did not appear to account for smaller ligand **2b**. The “acetyl” moiety of **1a** would clash with Glu294, even in the relatively open subunit A. The closed active site of subunit B would enforce even more stringent steric constraints on the bound ligand, which might be a partially disordered **2a** or **3a** instead of the modeled **4a**.

### Attempted trapping of acetylglutamyl anhydride with sodium borohydride

Borohydride can inactivate class I CoA-transferases supplied a valid substrate, by reducing an activated glutamate carboxylate thioester to 5-hydroxynorvaline (Solomon and Jencks, 1969). This has been done with AarC (Mullins et al., 2008). While both the acylglutamyl anhydride (internal carbonyl) or glutamyl-CoA thioester adducts could be inactivated, **1a** cannot form the latter. To test for anhydride formation, AarC and **1a** were incubated together for a week. Aliquots of the reaction mixture were withdrawn at intervals, mixed with sodium borohydride, and tested for residual SCACT activity. No loss of enzyme activity was observed in this experiment or in a control reaction lacking **1a** (Figure S2). This finding suggests that no anhydride adduct of AarC is present in solution, although the 5e5h structure suggests that one may be stabilized by the crystalline lattice.

### Decomposition of 1a and 2a

Direct detection of **1a** cleavage by analysis of small molecules formed in AarC crystal mother liquor was deemed to be infeasible. Instead, a **1a** stability assay was devised to detect the formation of truncated CoA analogs in reaction mixtures containing AarC. Analogs **2a** and **3a** were synthesized for use as authentic standards (Figure S3). HPLC chromatographic conditions that resolved **1a**, **1c**, and **2a** were identified (Figure S4). Under these conditions, the detection threshold for **2a** (in the presence of a ~20-fold molar excess of **1a**) was ~0.02 nmol.

The stability of **1a** was assessed by serial HPLC analysis of reaction mixtures incubated at ambient temperature. CoA analogs including **1a** were identified by retention time and **1a** was quantitated by comparing its peak area to a reference reaction mixture quenched immediately after the addition of enzyme.

First, **1a** was incubated in buffer without added enzyme. Under these conditions, **1a** was stable and no new peaks were observed in HPLC chromatograms, with 94–100% remaining after 168 h (Figure S5).

**Figure 7:**
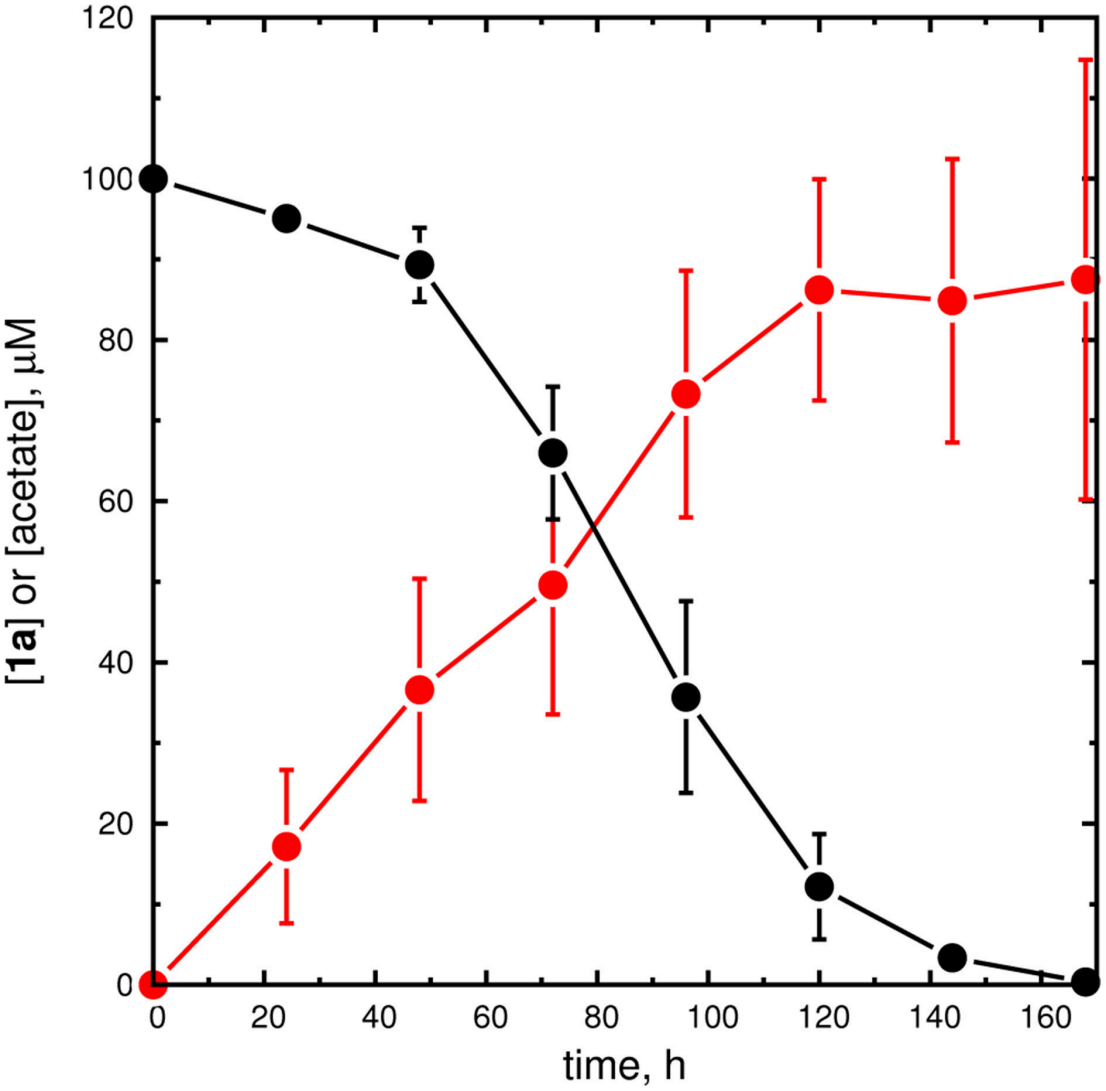
**Unfiltered reaction mixtures containing AarC decompose 1a**. Stability of **1a** in a reaction mixture initially containing 10 μM AarC and 100 μM **1a** at room temperature. Aliquots were withdrawn at the indicated time points and HPLC (**1a**, black filled circles), AK-ATPase assay (acetate, red filled circles), and MALDI-MS analyses were performed. The circles and error bars, respectively, depict average concentrations and standard deviations for three independent time courses. An alternative time course using ACS to detect acetate is shown in the Supplementary Material (Figure S13).

Second, **1a** and AarC were incubated together at a 10:1 molar ratio (subunit concentration). Delayed but complete loss of the HPLC peak corresponding to **1a** was observed (Figure 7). A parallel experiment monitoring the absorbance spectrum of crude reaction mixtures showed a progressive decrease at 260 nm (Figure S5), consistent with disintegration of the adenine moiety.

Third, **1a** and the inactive mutant AarC-E294A were incubated together at a 10:1 molar ratio. Delayed but incomplete loss of the HPLC peak corresponding to **1a** was observed (Figure S5), with ~37% remaining after 168 h. Therefore, active enzyme was not required for **1a** degradation.

Fourth, a reaction mixture containing **1a** and AarC, using the same conditions as the second experiment, was sterile-filtered shortly after mixing. After 168 h, 98% of the original **1a** was recovered, demonstrating that it is stable under these conditions. Furthermore, this result suggested that **1a** truncation is a microbe-mediated process.

A new peak observed in HPLC traces coincided with rapid loss of **1a** (Figure S6A). This late-eluting peak was collected, analyzed by MALDI-TOF MS, and found to have m/z = 712.13, corresponding to an [M+H-P_i_]^+^ ion (expected m/z 712.20) (data not shown). This compound was tentatively assigned as 3′-dephospho-AcMX (**1b**), and it appeared to be preferentially formed from **1a** not **1c** (Figure S6B). Peaks corresponding to **2a**, **3a**, or a peak likely to correspond to **4a** were not detected by HPLC analysis of **1a** stability assay reaction mixtures.

One potential explanation for the failure to detect **1a**-derived **2a** in solution stability assays would be rapid degradation of **2a**. In unfiltered reaction mixtures containing AarC, authentic **2a** decomposed completely, with a lag ~24 h longer than that observed for **1a** (Figure S7). We concluded that rapid **2a** decomposition could explain the failure to detect it by HPLC.

MALDI-TOF analysis of **1a** stability assay reaction mixtures was used to detect **1a**-derived compounds. Spectra acquired over 168 h showed an increasing proportion of species with smaller *m*/*z* values (Figure S8) that did not produce the same daughter ions as authentic **1a** (Figure S9) or **2a** (Figures S10, S11). We conclude that, if progressive shortening of the **1a** or **1b** aminopentanone moiety occurs, many or all subsequent degradation steps are rapid and appear to prevent accumulation of detectable levels of chain-shortened intermediates.

As a preliminary test of whether **1b** is an obligatory intermediate in **1a** decomposition, CoaE (dephospho-CoA kinase) and ATP were added to a **1a** stability assay. After 196 h, 98% of the original **1a** was recovered, compared to 27% in a reaction mixture lacking CoaE and ATP, and 105% of a no-enzyme control. The protective effect of CoaE, together with kinetic evidence that **1c** is a non-preferred substrate (Figure S6), is most consistent with **1a** dephosphorylation to **1b** as the first step in **1a** decomposition.

### Identification of 1a-derived acetate

Acetate from an unknown source was tentatively detected in the crystal structure of AarC crystallized with **1a**. Removal of the terminal two carbon atoms from **1a** (or **1b**) might result in the formation of acetate. On the basis of this consideration, we performed two sets of experiments intended to (1) unambiguously identify acetate as a product of **1a** degradation and (2) maximize acetate recovery, to estimate the stoichiometry of acetate production from **1a**.

In the first set of experiments, ACS was used to convert acetate to AcCoA, which was in turn positively identified by HPLC retention time and quantitated by comparison to an authentic AcCoA standard (Figure S12). An acetate standard solution was used to determine that this assay method recovers 92% of the original acetate as AcCoA (data not shown), with losses presumed to originate from sample processing and transfer steps. After 168 h, ~40 μM acetate was produced, corresponding to a 40% yield (uncorrected) relative to the initial **1a** concentration (Figure S13). No other acyl-CoA peaks were detected in HPLC chromatograms. This experiment is highly specific for acetate and places a *lower* limit on the stoichiometry of acetate recovered from **1a** breakdown.

In the second set of experiments, AK was used to convert acetate and ATP to acetyl phosphate and ADP, which was quantitated using a standard PK/LDH coupled ATPase assay. A set of standards demonstrated quantitative recovery of acetate, a detection limit of 0.1 nmol acetate (1–2% of the initial **1a**, and linearity to at least 100 μM acetate; Figure S14). Decomposition reaction mixtures analyzed with a no-AK control showed negligible NADH oxidation. Acetate was detected after 20 h of **1a** incubation and reached a maximum at 168 h, corresponding to a 88% yield relative to the initial **1a** concentration. As a one-pot assay, this assay minimizes sample losses but does not rule out the possibility of uncoupled ATP hydrolysis or that alternate substrates for coupling enzymes are produced during **1a** breakdown. This experiment therefore places an *upper* limit on the stoichiometry of acetate recovered from **1a** breakdown.

To identify the source of acetate, **2a** (100 μM) was allowed to degrade and products were analyzed using the quantitative (AK-coupled) method. After 168 h, 80% of the **2a** was gone, but little or no acetate was produced: ~5 μM, or 5% of the initial [**2a**]. Given an uncertainty of perhaps 10 μM, the evidence from this preliminary experiment indicates that **2a** does not serve as a source of acetate. We therefore infer that the near-stoichiometric conversion of **1a** to acetate requires a microbe-mediated cleavage of the aminopentanone moiety, and may involve excision of the terminal two-carbon unit.

### Crystallization of AarC with acetate

Acetic acid or acetate (collectively “acetate”) has been observed in several AarC structures without being provided in the crystallization solution. *A. aceti* normally contains high levels of cytoplasmic acetate (Menzel and Gottschalk, 1985; Steiner and Sauer, 2003), which is therefore likely to be the predominant protein-associated small anion. We crystallized AarC with exogenous acetate to identify potential binding sites for **1a**-derived acetate (PDB entry 5dw4). The final model contained three acetate binding sites in each subunit, related by the pseudo-twofold axis.

The first acetate binding site is near the active site and has been observed to bind acetate formed by AcCoA hydrolysis (PDB entry 4eu6 subunit B). Acetate (ACT 606A and 603B) accepts hydrogen bonds from the side chains of Ser71, Thr94, and Arg228 (Mullins and Kappock, 2012). This is the only acetate binding site not located at the rather polar interface between subunits.

The second acetate binding site overlaps a chloride binding site (CL 515) observed in earlier structures and is located on the flanks of the dimer. Acetate (ACT 605A and ACT 605B) accepts hydrogen bonds from the side chains of Asn112, Arg120, and Asn125 of the same subunit and the backbone of Gly443′ (the prime denotes a residue from the partner subunit).

The third acetate binding site overlaps the other chloride binding site (CL 516) observed in earlier structures and is located near the pseudo-twofold axis on the flat “top” of the dimer. Acetate (ACT 607A and 601B) accepts hydrogen bonds from the side chains of Arg354 (bidentate) and Arg354′ (monodentate) and the backbone NH of Val196′. Protein atoms in the interfacial acetate and chloride binding sites are nearly superimposable, indicating that the acetate displaces chloride ions supplied by the buffers used to isolate and crystallize recombinant AarC(H6).

Acetate crystals lacked the buffer-derived citrate ligands observed in earlier “open” structures (PDB entries 4eu3, 4eu7, and 4eud). As anticipated, each subunit in the AarC•acetate complex possesses active site parameters typical of other open conformations (Figure 3).

### Crystallization of AarC with 2a

Crystallography cannot unambiguously identify **2a** as the ligand in AarC crystals grown with **1a** (denoted AarC+**1a**). The putative **2a** propyl sidechain has relatively high B-factors, as expected for a flexible, nonpolar group in an open, polar active site (PDB entry 5e5hA). AarC was crystallized with synthetic **2a** to compare an authentic AarC•**2a** complex with the AarC+**1a** structure. AarC•**2a**•acetate crystals were grown either with acetate present in the initial crystallization solution or with acetate added long after AarC•**2a** crystals formed. Several X-ray data sets were collected using crystals from drops that initially contained either **2a** or **2a**+acetate; all diffracted to 1.66 Å resolution. A better data set (PDB entry 5dw6) was obtained from a crystal that was grown in the presence of **2a** prior to the addition of acetate.

The AarC•**2a** complex (PDB entry 5dw5) contained three chloride ions, one near the pseudo-twofold axis and two on the flanks of the dimer. One of the latter chlorides (CL 601A) and an acetate ligand (ACT 601B) were located so close together we assumed that they could not be simultaneously present. The final refined fractional occupancies were 63 and 37%, respectively. The acetate orientation was different from that observed in the AarC•acetate complex (PDB entry 5dw4): it accepted a hydrogen bond from the side chain of Asn112A. Since acetate was not intentionally added to this crystal, we considered but ultimately discarded the possibility that a formate was carried over from the isolation of **2a**. Other aspects of the structure were essentially the same as described next for acetate-soaked crystals, other than replacing chloride ions associated with two different sites in each subunit (Mullins and Kappock, 2012). An acetate-capped, solvent-filled tunnel provides a possible path for entry of two buried acetates near the pseudo-twofold axis.

**Figure 8:**
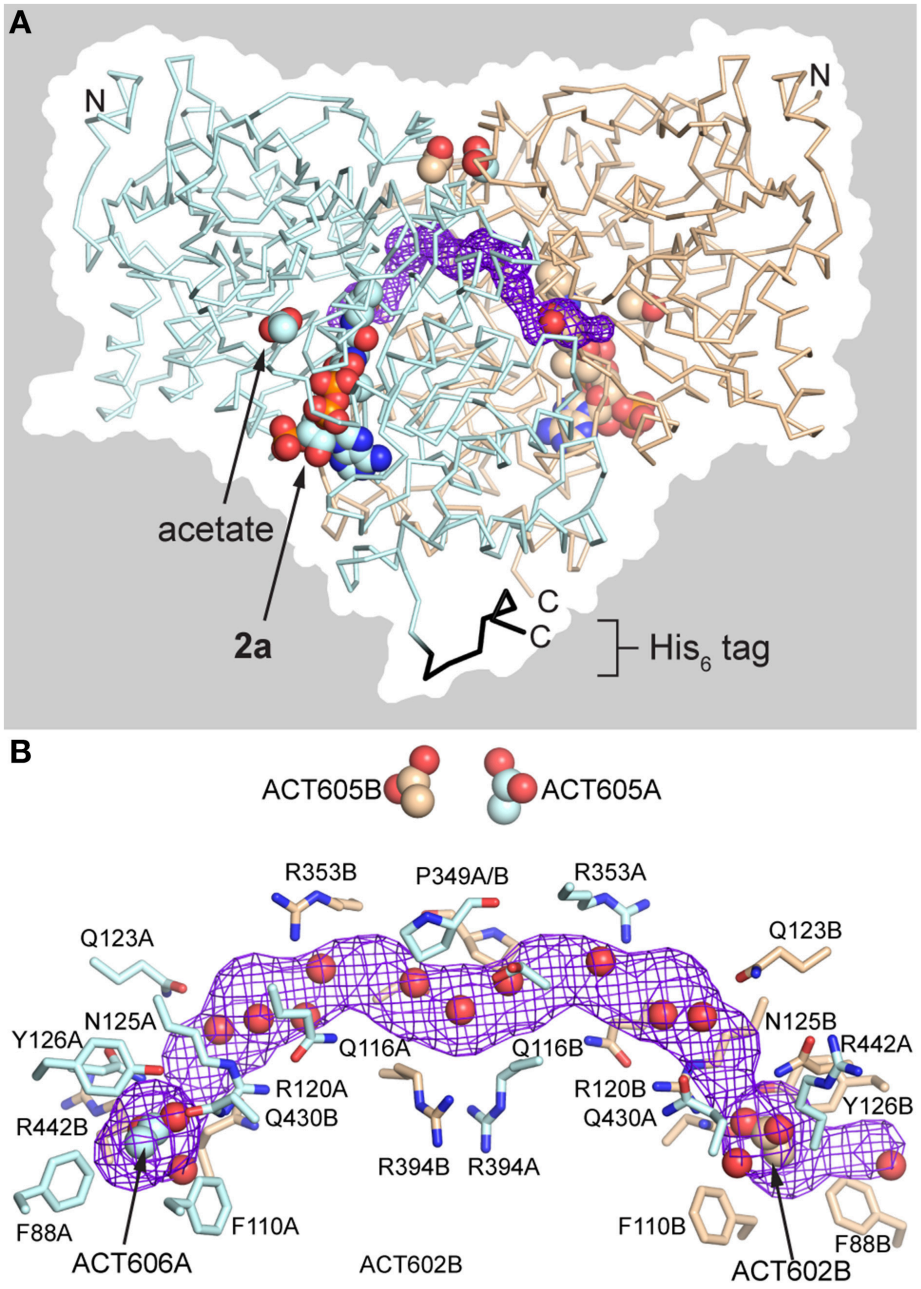
**Anion-plugged tunnel in AarC•2a•acetate complex (PDB entry 5dw6)**. **(A)** View of the dimer with acetate and **2a** rendered as spheres in each subunit. The backbone is shown in ribbons rendering: blue, subunit A; black, subunit A His_6_ tag; tan, subunit B. The tunnel that runs along the subunit interface (purple mesh), running from the left rear to the right front, is bisected by the vertical pseudo-twofold axis. The dimer surface is shown in silhouette. **(B)** Longitudinal view of the interface tunnel, rotated around the pseudo-twofold axis by ~60° from the view in panel A. The tunnel (purple mesh) is defined by residue side chains that are depicted in sticks and backbone atoms (not shown). All atoms of both Pro349 residues are shown near the pseudo-twofold axis at center. The polar side chains depicted are generally involved in buried salt-bridges or hydrogen bonding interactions. The flank-binding acetates and ordered waters within 1.4 Å of the midpoint of the tunnel are depicted as spheres.

The AarC•**2a**•acetate complex (PDB entry 5dw6) contained **2a** and an acetate in each active site, with four acetate ligands at the subunit interface (two on the flanks of the dimer and two at the pseudo-twofold axis). Subunits A and B adopt the open and closed conformations, respectively (Figure 3). An in-plane ~120° rotation of the active-site acetate ligand in subunit B, relative to previous orientations (e.g., PDB entry 4eu6), gave a slightly better fit to the data. This may be related to the exclusion of the carboxylate-binding residue Arg228B from the closed active site. A bow-shaped, 65 Å long, narrow (average width ~2 Å), and hydrophilic tunnel was plugged by the two flank-binding acetates that supplant chloride ions (Figure 8). Eight basic and zero acidic residues line the tunnel, but only the acetate binding sites have a substantial positive charge. Crystals containing **2a**, including those grown without added acetate, did not contain ordered citrate ligands, even though subunit A adopts an open conformation and the crystallization conditions were, apart from CoA, identical to those that yielded AarC(H6) crystals with a citrate in each active site (PDB entries 4eu7 and 4eud).

**Figure 9:**
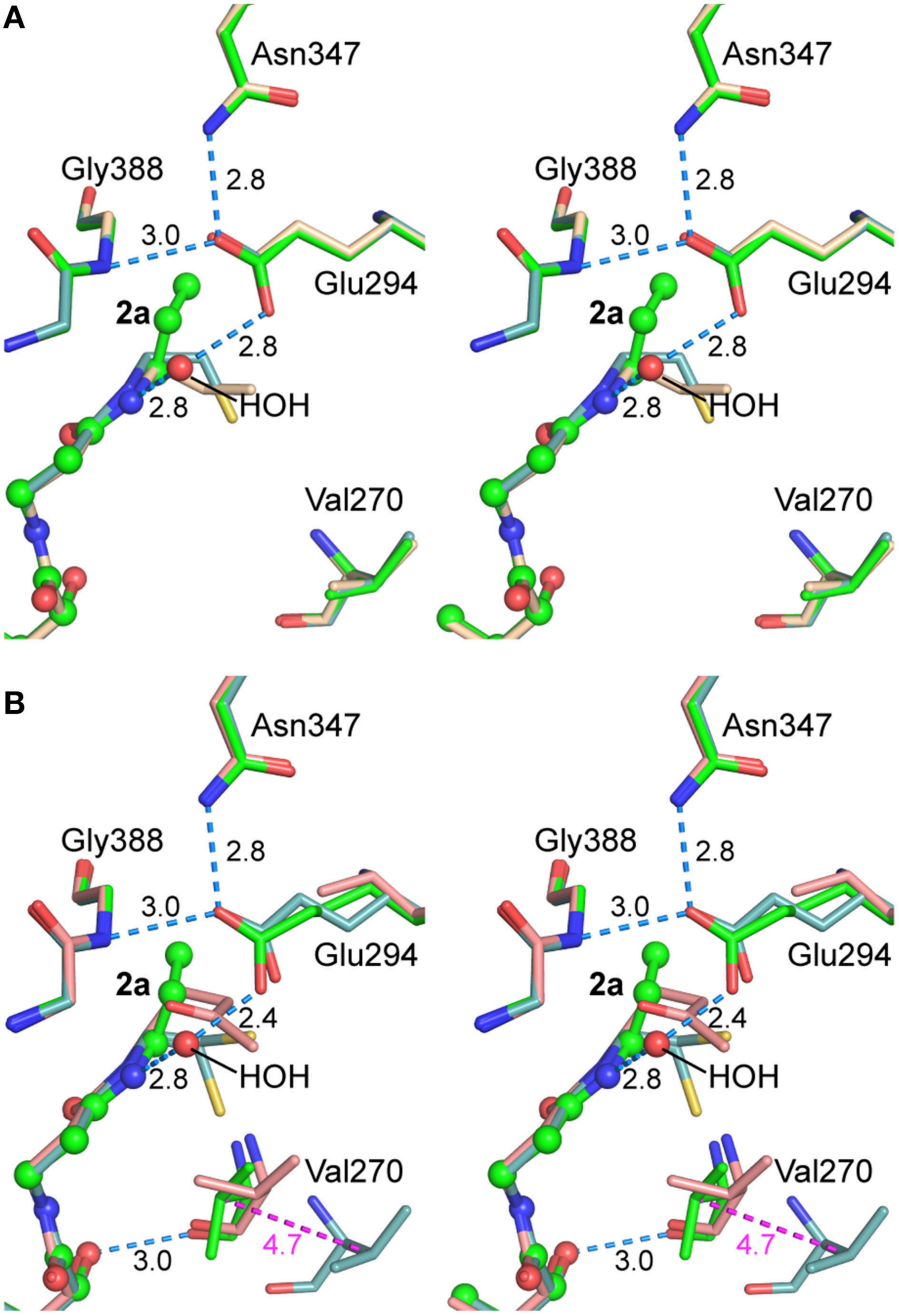
**Stereograms of the AarC•2a•acetate active sites**. **(A)** Subunit A, in the open conformation for all structures depicted. Carbon atoms in superposed B subunits are green in AarC•**2a**•acetate (PDB entry 5dw6; spheres are shown for **2a** and HOH 923A), wheat in AarC+**1a** (PDB entry 5e5h), and light blue in AarC•citrate (PDB entry 4eu7). **(B)** Subunit B, in the closed conformation except where indicated. Carbon atoms in superposed B subunits are green in AarC•**2a**•acetate (PDB entry 5dw6; spheres are shown for **2a** and HOH 713B), salmon in AarC-E294A•**1a** (PDB entry 4euc), and light blue in AarC•citrate (PDB entry 4eu7, open conformation). Distances (in Å) are shown for 5dw6 hydrogen bonds and the shift of Val270B CB from the open to closed conformation (magenta). The orientation is the same as in Figure 4.

The CoA analog **2a** binds in the same orientation in the two active sites (Figure 9), which is notable because one is open (subunit A) and the other is closed (subunit B). In the fully closed conformation, a hydrogen bond was observed between Val270 carbonyl and the OAP hydroxyl in the pantoic acid moiety of **2a**. Relative to the AarC+**1a** structure (PDB entry 5e5h), the **2a** propyl group is closer to a fully extended conformation; the orientations differ by a 107° rotation about the C2P–C3P bond. The terminal group that is modeled as a methyl in the **1a**-derived molecule has a close contact with the nearest carboxylate oxygen atom in Glu294A (3.1 Å). In contrast, the structure containing authentic **2a** adopts an almost perpendicular conformation that buries the terminal methyl in a largely hydrophobic region ~4 Å from Gln267A CG, Glu294A CG, Gly388 CA, and the Phe392 phenyl ring. Density associated with the 3′ phosphoryl was unambiguous for the AarC•**2a**(•acetate) structure but not for the **1a**-derived molecule. In summary, AarC crystals grown with chemically defined ligands including **2a** did not recapitulate the structure obtained with **1a** under conditions associated with its decomposition. This likely arises from differences in crystallization kinetics and conditions or the presence of different ligands. We favor the latter as a working hypothesis, as the terminus of the **1a**-derived ligand appears to be more polar and perhaps somewhat larger than the aminopropyl group in **2a**.

Attempts to crystallize AarC with **3a**, with and without exogenous acetate, yielded only clear drops devoid of crystals.

## Discussion

Enzyme substrates that incorporate large cofactors, such as acyl-CoAs, form extensive protein-ligand interfaces that can increase substrate specificity, enzyme reaction rates, and thereby metabolic flux. For example, bacterial biosynthetic enzymes recognize NADPH, against a 20-fold excess of NAD^+^ (Bennett et al., 2009), using its remote 3′-phosphate. Nonreactive regions of a large substrate contribute “intrinsic binding energy” that enzymes such as the CoA-transferases may exploit in catalysis (Jencks, 1975). Jencks and co-workers showed that remote interactions further accelerate successive acyltransfer and thiolysis reactions (Moore and Jencks, 1982) initiated by tight clamping of the acyl-CoA thioester by the enzyme (White and Jencks, 1976). This prediction was later confirmed by crystal structures showing progressive constriction of the AarC active site (Mullins and Kappock, 2012).

Analog studies show that the ADP and pantoate moieties of CoA have opposing effects on glutamyl-CoA thioester stability: the nucleotide supplies binding energy used to pull the pantoate moiety into the active site and form a thioester whereas subsequent interactions between the enzyme and pantoate moiety are destabilizing and increase thioester reactivity toward carboxylates (Whitty et al., 1995). A substrate “split into pieces” can sometimes form an alternative Michaelis complex that undergoes chemical conversion (Amyes and Richard, 2013). Class I acyl-CoA transferases, however, fail this test: only a covalently linked ADP moiety confers a rate increase (Fierke and Jencks, 1986). This suggests that substrate conformational dynamics and mechanical coupling within the ligand are important aspects of harnessing binding affinity to achieve catalytic rate accelerations.

Analog studies have not yet explained how CoA-transferases productively engage valid acyl-CoA substrates but discriminate against unproductive interactions with free CoA, which possesses nearly all of the same features (Fierke and Jencks, 1986). Most interactions of free CoA with the open conformation of AarC are mediated by waters that are squeezed out when the active site closes, with a corresponding gain in entropy (Mullins and Kappock, 2012). One practical advantage of this inability to close the active site, and thereby assemble a functional active site, is that free CoA is not a strong enzyme inhibitor (Blair, 1969); inhibition of AarC by CoA is competitive (*K*_i_ = 16 μM) and comparable to acyl-CoA substrate *K*_M_ values (Mullins, 2012). In addition to the important role the “viselike grip” exerted in the immediate vicinity of the acyl-CoA thioester has on catalysis (White and Jencks, 1976), we suggest that AarC, and by extension other class I CoA-transferase active sites, positively recognizes free CoA to prevent the final stage of active site closure.

### Distinguishing CoA from Acyl-CoA

Conformational dynamics regulate CoA-transferase chemistry, since only completely closed complexes contain both a complete external oxyanion hole and properly oriented Val270, which are needed to initiate reactions with acyl-CoA substrates. A key to furthering the understanding of CoA-transferases is therefore to generate crystals containing closed active sites, using mutant enzymes or substrate analogs that cannot undergo a complete enzymatic reaction.

In our first set of AarC structures (Mullins and Kappock, 2012), we observed complete active site closure but only in complexes of mutants with, among other effects, enlarged active site pockets (S71A or E294A in PDB entries 4eu8B, 4eub, and 4euc; Figure 3). The most closed structure of wild-type AarC bound to CoA (PDB entry 4eu5B) shows Val270 in a closed conformation, Arg228 in the open (inward) position, and the 230s loop in an intermediate location (Figure 3). Crystal-packing interactions characteristic of the subunit B active site may bias the protein conformation and favor formation of a Val270 amide-CoA thiolate contact.

In the current work, we have used substrate analogs to trap closed complexes. While co-crystallization of AarC with **1a** gave ambiguous results, the attempt to reproduce this complex with authentic **2a** yielded the first completely closed complexes of the wild-type enzyme (PDB entries 4dw5B and 4dw6B; Figure 3). The position of Glu294, buttressed by Asn347, remains largely unchanged in the closed complex, lending further support to the idea that enzyme closing exerts mechanical forces that favor attack of the nucleophile on the acyl-CoA substrate (inset, Figure 1). Other polar groups within the active site, such as the mobile Val270 residue, occupy locations that are consistent with roles in catalysis proposed previously. Moreover, these residues adopt similar orientations in crystal structures of mutant AarC complexes. By pre-positioning the Val270 amide that is proposed to stabilize the CoA thiolate leaving group, the enzyme facilitates reactions involving the key acylglutamyl anhydride intermediate.

In PDB entry 4eu6A, the CoA thiol(ate) adopts a near-attack position equidistant from the external and internal carbonyl carbon atoms of a trapped acetylglutamyl anhydride. In **2a** complexes, an inward rotation of the C2P-C3P bond moves the corresponding methyl terminus into a small pocket in both open (PDB entries 4dw5A and 4dw6A) and closed (PDB entries 4dw5B and 4dw6B) complexes (Figure 4). A CoA sulfur forced into this pocket would encounter electrostatic clashes with electron-rich carbonyl oxygen atoms in Gly388 and the Glu294 carboxylate. The **2a** terminus is rotated by ~120° relative to the outward-facing CoA thiol(ate) observed in open active sites and the mostly closed active site (PDB entry 4eu5B). In this location, the **2a** methyl terminus appears unlikely to affect active site conformational dynamics. A detailed comparison shows that other regions of bound CoA and **2a** adopt the same conformations. Similarly, there are few differences in the protein region between CoA and the surface-located 230s loop that might account for the more open conformation of the latter.

The principal difference seems to be whether the ligand makes a polar contact with the Glu294 carboxylate, an interaction that we propose “props open” the active site and inhibits the final steps in active site closure. In PDB entry 4eu5B, the outwardly rotated CoA sulfur atom is 2.9 Å from the closest Glu294B carboxylate oxygen atom, 3.4 Å from the Val270 nitrogen atom, and 12 Å from the closest guanidinium nitrogen atom of the inward-facing Arg228B. Since a CoA thiolate could not form so close to a carboxylate, a CoA thiol is likely present and may be stabilized by a hydrogen bond involving Glu294. No such interaction is possible in the fully closed complexes observed for AarC-E294A•CoA (PDB entry 4eub), AarC-E294A•**1a** (PDB entry 4euc), or AarC•**2a** (PDB entries 4dw5B and 4dw6B). Acyl-CoA substrates also lack an appropriate hydrogen bond donor.

Maintaining CoA at the proper cytoplasmic concentration and relative ratio to the acyl-CoA pool allows effective carboxylate utilization and regulates flux through central metabolism (reviewed recently by Hentchel and Escalante-Semerena, 2015). The ability to positively recognize CoA and prevent diversion of its binding energy into the formation of a dead-end closed complex would therefore confer an evolutionary advantage on diverse CoA-transferases. Positive and highly specific selection for CoA binding could also explain why oxy-CoA is more potent than CoA as an inhibitor of mammalian succinyl CoA:3-ketoacid CoA-transferase (Jencks, 1973).

The mechanism by which a single polar contact might prevent the distant 230s loop from fully closing may involve protein-ligand dynamics sensitive to the CoA thiol-carboxylate contact. The failure of “split substrates” to support efficient CoA-transferase catalysis similarly implicates dynamic effects of an intact CoA moiety as a regulator of the open/closed protein conformational equilibrium.

### Reinforcement of Glu294 by internal oxyanion hole residue Asn347

Oxyanion holes are typically rigid features tasked with precise dipole positioning (Kamerlin et al., 2010). The AarC-N347A mutant was designed to perturb the internal oxyanion hole and thereby to slow enzyme chemistry, which is not rate-limiting in wild-type enzyme steady-state kinetics (White and Jencks, 1976). A relatively high residual activity (Mullins and Kappock, 2012) suggested that an additional active site water might restore the function of the mutated internal oxyanion hole. Ordered waters observed near the site of each missing carboxamide appear to confirm this suspicion, which indicates that AarC-N347A cannot be used to completely stop CoA thiolysis nor preferentially stabilize acylglutamyl anhydride intermediates.

The AarC-N347A crystal structure, however, suggests that Asn347 affects the reactivity of Glu294 in its multiple roles as a nucleophile, as a part of internal oxyanions, and as the anchor point for external oxyanions. Without Asn347, Glu294 moves from a position suitable for nucleophilic attack on an acyl-CoA substrate by a carboxylate oxygen *syn* lone pair to one better aligned for attack by a less-basic and less-nucleophilic *anti* lone pair (Gandour, 1981; Li and Houk, 1989). Since AarC-N347A appears to favor the closed conformation, its effect on catalysis might also stem from impeded acyl-CoA product dissociation during steady-state turnover. We disfavor this alternative hypothesis since all four AarC-N347A *K*_M_ values are similar to wild-type AarC *K*_M_ values (Mullins and Kappock, 2012). While these findings are consistent with a prior hypothesis that the N347A mutant primarily affects enzyme-mediated, unimolecular chemical steps, it now seems more likely that reactions at both anhydride carbonyls are affected, not just those involving the internal carbonyl. Our working model is therefore that Asn347 polarizes and positions Glu294, aiding in its roles as nucleophile and, potentially, as the leaving group in the CoA thiolysis reaction.

Two large families within the class I CoA-transferase superfamily have different, highly conserved carboxamide residues, which suggests that the internal oxyanion hole is more tolerant of variation than the strictly conserved external oxyanion hole. One potential reason for this difference is that the internal oxyanion hole is preorganized whereas part of the external oxyanion hole is mobile, delivered by the CoA itself, and must adopt the correct configuration prior to or during active site closure (Mullins and Kappock, 2012). In addition, the covalent attachment of Glu294 to the rigid C-terminal β-sheet may limit the intrinsic flexibility of the anhydride internal carbonyl, thereby increasing its reactivity relative to the distal external carbonyl.

### A working hypothesis for 1a cleavage

The class I CoA-transferase external oxyanion hole is composed of two amide dipoles, the backbone nitrogen of Gly388 and the distal amide nitrogen of the substrate itself (Mullins and Kappock, 2012). We hypothesized that **1a**, a competitive inhibitor that binds tightly to AarC (*K*_i_ = 17 μM, *K*_d_ = 0.8 μM; Mullins et al., 2008), could be used to trap a stable hemiacetal analog of the external oxyanion in a fully closed active site. This experiment was thwarted by unexpected microbe-mediated truncation of the substrate analog, which ultimately resulted in complete consumption of the organic ligand (Attempts to crystallize the hemiacetal complex under sterile conditions are ongoing).

Since **1a** degradation is slow and follows a long lag period, it was not previously detected during routine activity assays but it was remarkably reproducible: **1a** degradation was observed in eight out of nine trials with independently purified batches of AarC or an inactive mutant, indicating that **1a** cleavage has nothing to do with AarC activity. We speculate that the lag in **1a** degradation is due to the induction of microbial enzymes, including the postulated 3′-phosphatase.

We prepared AarC•**2a** crystals in an attempt to positively identify the **1a**-derived species, but instead obtained a fully closed complex (discussed above) that was distinctly different from the partly open complex obtained from AarC and **1a**. One explanation might be the presence of a 3′-phosphate moiety in **2a**, which was missing in at least one subunit of the structure derived from AarC and **1a**. We discount this explanation, given that the 3′-phosphate is largely solvent-exposed and prior work that indicates that it has little influence on substrate affinity (Whitty et al., 1995; Mullins and Kappock, 2012). A more likely explanation for the difference is the absence of an acetylglutamyl anhydride from the AarC•**2a** crystals; crystals obtained from AarC+**1a** adopt an intermediate conformation characteristic of other covalent adducts (Figure 3, lower right quadrant) that may protect the labile anhydride. Indeed, the absence of a CoA thiolate in **2a** would facilitate anhydride hydrolysis, particularly in solution, which could explain why the borohydride-trapping treatment failed to detect anhydride adducts.

**Figure 10:**
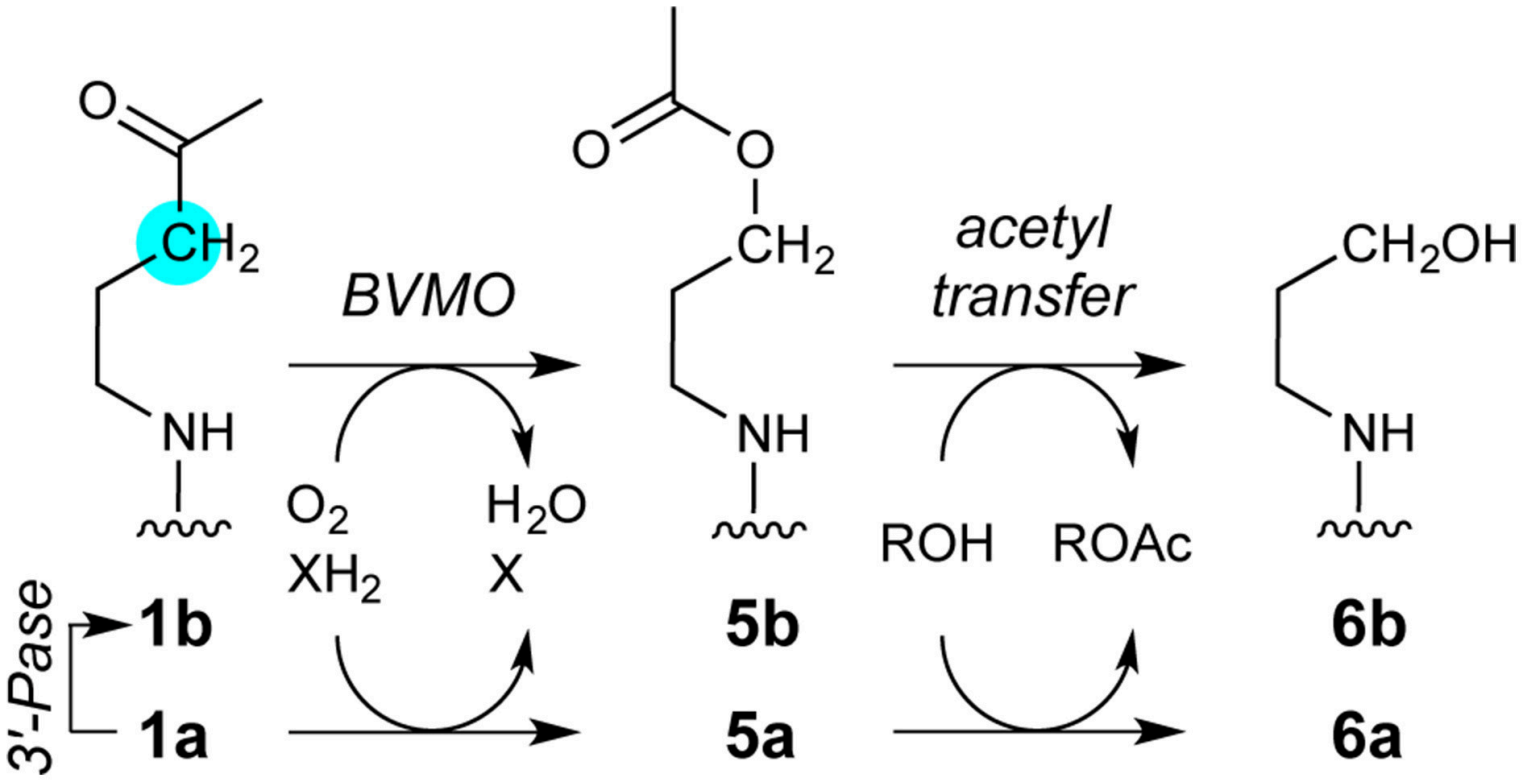
**Speculative pathway for 1a degradation by microbial enzymes**. The key step would be oxidation of the **1a** ketone carbonyl by a Baeyer-Villiger monooxygenase (BVMO), yielding **5a**. Unlike **1a**, **5a** contains an activated acetyl moiety capable of producing both an acetylglutamyl anhydride adduct (Figure 6) and free acetate (Figure 7). The other product of acyl transfer, **6a**, appears to be degraded rapidly by undefined enzymes. The intermediate detection of **1b** suggests that a 3′-phosphatase (3′-Pase) may compete with, or form the substrate for, BVMO. Ions consistent with transient production of **5b**, **6a**, or both during **1a** decomposition were detected by MALDI-TOF (Figure S8). Mass spectrometric evidence supporting parallel pathways for **1a** degradation is detailed in the Supplementary Material (Figure S15).

The observation of an acetylglutamyl anhydride in the crystalline lattice (PDB entry 5e5h) and the direct detection of acetate production in solution from **1a** but not **2a** indicate that the **1a** ketone is converted to an activated acetyl donor. Oxidation of **1a** or a degradation product by at least one microbial enzyme provides a straightforward and concise, albeit speculative, route to an activated acetyl group: a Baeyer-Villiger monooxygenase (BVMO) acting on **1a** could yield ester **5a** (Figure 10). Short-chain alkyl methyl ketones that resemble the aminopentanone terminus of **1a** are converted to acetate esters by a *Pseudomonas* BVMO (Onaca et al., 2007).

Intriguingly, while **5a** resembles AcCoA and would be well-suited to acylate Glu294, the product alcohol **6a** (Figure 10) could not perform subsequent steps in the AarC reaction. For example, **6a** would be less nucleophilic than CoA, which might selectively stabilize the acetylglutamyl anhydride and account for its detection by crystallography. The near-stoichiometric appearance of acetate and disappearance of **1a** in solution studies is also consistent with irreversible conversion of **1a** to an activated acetyl donor (Figure 7).

The terminus of the **1a**-derived CoA analog, presently modeled as an alkyl chain (PDB entry 5e5h), occupies a more open active site and a more polar region than the terminus of authentic **2a**. These discrepancies might be explained if the former is actually **6a** with a partially disordered alcohol terminus.

Finally, the possibility that **5a** can form a long-lived anhydride intermediate suggests future experiments with authentic **5a** or *O*-acetyl oxyCoA, an analog with one fewer methylene. AarC acylating reagents like these may enable the stoichiometric preparation and trapping of fully closed, covalently modified enzyme active sites.

### Concluding remarks

This study has advanced our understanding of how AarC selects its substrates and activates them for a sophisticated multi-step reaction. We report here the first fully closed structure of wild-type AarC bound to a CoA analog, which shows that the key Glu294 does not move during active site closure. This finding supports the proposal that steric strain, introduced by active site closure, is employed to overcome kinetic and thermodynamic barriers to acylglutamyl anhydride formation. The internal oxyanion hole residue Asn347 appears to be an important buttress for Glu294. A crystal structure of the N347A mutant revealed unanticipated roles in positioning and tuning the reactivity of Glu294, using steric, electrostatic, and possibly stereoelectronic effects. Degradation of an AcCoA analog by unidentified microbial enzymes produced an activated acetyl donor and acetate, which suggests that ester analogs of AcCoA (e.g., **5a**) could be used to produce a stabilized acetylglutamyl anhydride on AarC. Deletion of a thiol-carboxylate contact between CoA and AarC Glu294 appears to disrupt a “safety catch” mechanism that prevents inappropriate capture of CoA but allows full active site closure onto valid acyl-CoA substrates. This mechanism would enable AarC and other CoA-transferases to exploit favorable, remote interactions with a large substrate to accelerate unfavorable reactions while avoiding unproductive formation of a tight CoA complex.

## Author contributions

JM and EM prepared reagents, grew crystals, and collected and analyzed biochemical data. TK and EM collected and analyzed crystallographic data. JM, EM, and TK interpreted data and wrote the manuscript. TK supervised the project.

### Conflict of interest statement

The authors declare that the research was conducted in the absence of any commercial or financial relationships that could be construed as a potential conflict of interest.

## References

[B1] Adams P. D. Afonine P. V. Bunkóczi G. Chen V. B. Davis I. W. Echols N. (2010). PHENIX: a comprehensive Python-based system for macromolecular structure solution. Acta Crystallogr. D Biol. Crystallogr. 66, 213–221. 10.1107/S0907444909052925 20124702 PMC2815670

[B2] Amyes T. L. Richard J. P. (1992). Generation and stability of a simple thiol ester enolate in aqueous solution. J. Am. Chem. Soc. 114, 10297–10302. 10.1021/ja00052a028

[B3] Amyes T. L. Richard J. P. (2013). Specificity in transition state binding: the Pauling model revisited. Biochemistry 52, 2021–2035. 10.1021/bi301491r 23327224 PMC3679207

[B4] Bennett B. D. Kimball E. H. Gao M. Osterhout R. Van Dien S. J. Rabinowitz J. D. (2009). Absolute metabolite concentrations and implied enzyme active site occupancy in *Escherichia coli*. Nat. Chem. Biol. 5, 593–599. 10.1038/nchembio.186 19561621 PMC2754216

[B5] Berman H. Henrick K. Nakamura H. (2003). Announcing the worldwide protein data bank. Nat. Struct. Biol. 10:980. 10.1038/nsb1203-980 14634627

[B6] Blair J. B. (1969). Skeletal muscle coenzyme A transferase. Purification and properties. J. Biol. Chem. 244, 951–954. 4976792

[B7] Bürgi H. B. Dunitz J. D. Shefter E. (1973). Geometrical reaction coordinates. II. Nucleophilic addition to a carbonyl group. J. Am. Chem. Soc. 95, 5065–5067. 10.1021/ja00796a058

[B8] Burns K. L. Gelbaum L. T. Sullards M. C. Bostwick D. E. May S. W. (2005). Iso-coenzyme A. J. Biol. Chem. 280, 16550–16558. 10.1074/jbc.M411898200 15708855

[B9] Calder R. B. Williams R. S. Ramaswamy G. Rock C. O. Campbell E. Unkles S. E.. (1999). Cloning and characterization of a eukaryotic pantothenate kinase gene (panK) from *Aspergillus nidulans*. J. Biol. Chem. 274, 2014–2020. 10.1074/jbc.274.4.2014 9890959

[B10] Chen V. B. Arendall W. B. III Headd J. J. Keedy D. A. Immormino R. M. Kapral G. J. (2010). MolProbity: all-atom structure validation for macromolecular crystallography. Acta Crystallogr. D Biol. Crystallogr. 66, 12–21. 10.1107/S0907444909042073 20057044 PMC2803126

[B11] DeLano W. L. (2002). The PyMOL Molecular Graphics System. Palo Alto, CA. Avaliable online at: http://www.pymol.org

[B12] Emsley P. Lohkamp B. Scott W. G. Cowtan K. (2010). Features and development of Coot. Acta Crystallogr. D Biol. Crystallogr. 66, 486–501. 10.1107/S0907444910007493 20383002 PMC2852313

[B13] Ferry J. G. (2011). Acetate kinase and phosphotransacetylase. Methods Enzymol. 494, 219–231. 10.1016/B978-0-12-385112-3.00011-1 21402217

[B14] Fierke C. A. Jencks W. P. (1986). Two functional domains of coenzyme A activate catalysis by coenzyme A transferase. Pantetheine and adenosine 3′-phosphate 5′-diphosphate. J. Biol. Chem. 261, 7603–7606. 3458707

[B15] Francois J. A. Starks C. M. Sivanuntakorn S. Jiang H. Ransome A. E. Nam J.-W.. (2006). Structure of a NADH-insensitive hexameric citrate synthase that resists acid inactivation. Biochemistry 45, 13487–13499. 10.1021/bi061083k 17087502

[B16] Furdui C. M. Poole L. B. (2014). Chemical approaches to detect and analyze protein sulfenic acids. Mass Spectrom. Rev. 33, 126–146. 10.1002/mas.21384 24105931 PMC3946320

[B17] Gandour R. D. (1981). On the importance of orientation in general base catalysis by carboxylate. Bioorg. Chem. 10, 169–176. 10.1016/0045-2068(81)90020-1

[B18] Hentchel K. L. Escalante-Semerena J. C. (2015). Acylation of biomolecules in prokaryotes: a widespread strategy for the control of biological function and metabolic stress. Microbiol. Mol. Biol. Rev. 79, 321–346. 10.1128/MMBR.00020-15 26179745 PMC4503791

[B19] Herscovitch M. Perkins E. Baltus A. Fan M. (2012). Addgene provides an open forum for plasmid sharing. Nat. Biotechnol. 30, 316–317. 10.1038/nbt.2177 22491276

[B20] Jencks W. P. (1973). Coenzyme A transferases, in The Enzymes, 3rd Edn., Vol. 9B, ed Boyer P. D. (New York, NY; Academic Press), 483–496.

[B21] Jencks W. P. (1975). Binding energy, specificity, and enzymic catalysis: the circe effect. Adv. Enzymol. Relat. Areas Mol. Biol. 43, 219–410. 10.1002/9780470122884.ch4 892

[B22] Kamerlin S. C. L. Chu Z. T. Warshel A. (2010). On catalytic preorganization in oxyanion holes: highlighting the problems with gas-phase modeling of oxyanion holes and illustrating the need for complete enzyme models. J. Org. Chem. 75, 6391–6401. 10.1021/jo100651s 20825150 PMC2945449

[B23] Kapinos L. E. Operschall B. P. Larsen E. Sigel H. (2011). Understanding the acid-base properties of adenosine: the intrinsic basicities of N1, N3 and N7. Chem. Eur. J. 17, 8156–8164. 10.1002/chem.201003544 21626581

[B24] Kleywegt G. J. (2007). Crystallographic refinement of ligand complexes. Acta Crystallogr. D Biol. Crystallogr. 63, 94–100. 10.1107/S0907444906022657 17164531 PMC2483469

[B25] Li Y. Houk K. N. (1989). Theoretical assessments of the basicity and nucleophilicity of carboxylate syn and anti lone pairs. J. Am. Chem. Soc. 111, 4505–4507. 10.1021/ja00194a059

[B26] Lovell J. Simon C. Word J. M. Richardson J. S. Richardson D. C. (2000). The penultimate rotamer library. Proteins 40, 389–408. 10.1002/1097-0134(20000815)40:3<389::AID-PROT50>3.0.CO;2-2 10861930

[B27] Malabanan M. M. Amyes T. L. Richard J. P. (2010). A role for flexible loops in enzyme catalysis. Curr. Opin. Struct. Biol. 20, 702–710. 10.1016/j.sbi.2010.09.005 20951028 PMC2994964

[B28] Menzel U. Gottschalk G. (1985). The internal pH of Acetobacterium wieringae and *Acetobacter aceti* during growth and production of acetic acid. Arch. Microbiol. 143, 47–51. 10.1007/BF00414767

[B29] Merritt E. A. (2012). To B or not to B: a question of resolution? Acta Crystallogr. D Biol. Crystallogr. 68, 468–477. 10.1107/S0907444911028320 22505267 PMC3322606

[B30] Moore S. A. Jencks W. P. (1982). Formation of active site thiol esters of CoA transferase and the dependence of catalysis on specific binding interactions. J. Biol. Chem. 257, 10893–10907. 6955308

[B31] Mullins E. A. (2012). A Specialized Citric Acid Cycle Requiring Succinyl-Coenzyme A (CoA):Acetate CoA-Transferase (AarC) Confers Acetic Acid Resistance on the Acidophile Acetobacter aceti. Ph.D. thesis, Washington University in St. Louis, St. Louis, MO. 10.1128/JB.00405-08 PMC2447011 18502856

[B32] Mullins E. A. Francois J. A. Kappock T. J. (2008). A specialized citric acid cycle requiring succinyl-coenzyme A (CoA):acetate CoA-transferase (AarC) confers acetic acid resistance on the acidophile *Acetobacter aceti*. J. Bacteriol. 190, 4933–4940. 10.1128/JB.00405-08 18502856 PMC2447011

[B33] Mullins E. A. Kappock T. J. (2012). Crystal structures of *Acetobacter aceti* succinyl-coenzyme A (CoA):acetate CoA-transferase (AarC) reveal specificity determinants and illustrate the mechanism used by class I CoA-transferases. Biochemistry 51, 8422–8434. 10.1021/bi300957f 23030530

[B34] Onaca C. Kieninger M. Engesser K.-H. Altenbuchner J. (2007). Degradation of alkyl methyl ketones by *Pseudomonas veronii* MEK700. J. Bacteriol. 189, 3759–3767. 10.1128/JB.01279-06 17351032 PMC1913341

[B35] Otwinowski Z. Minor W. (1997). Processing of X-ray diffraction data collected in oscillation mode. Methods Enzymol. 276, 307–326. 10.1016/S0076-6879(97)76066-X 27754618

[B36] Read R. J. Adams P. D. Arendall W. B. III Brunger A. T. Emsley P. Joosten R. P.. (2011). A new generation of crystallographic validation tools for the protein data bank. Structure 19, 1395–1412. 10.1016/j.str.2011.08.006 22000512 PMC3195755

[B37] Sehnal D. Svobodová Vařeková R. Berka K. Pravda L. Navrátilová V. Banáš P.. (2013). MOLE 2.0: advanced approach for analysis of biomacromolecular channels. J. Cheminform. 5, 39. 10.1186/1758-2946-5-39 23953065 PMC3765717

[B38] Solomon F. Jencks W. P. (1969). Identification of an enzyme-γ-glutamyl coenzyme A intermediate from coenzyme A transferase. J. Biol. Chem. 244, 1079–1081. 5769180

[B39] Steiner P. Sauer U. (2003). Long-term continuous evolution of acetate resistant *Acetobacter aceti*. Biotechnol. Bioeng. 84, 40–44. 10.1002/bit.10741 12910541

[B40] Strauss E. Begley T. P. (2002). The antibiotic activity of N-pentylpantothenamide results from its conversion to ethyldethia-coenzyme A, a coenzyme A antimetabolite. J. Biol. Chem. 277, 48205–48209. 10.1074/jbc.M204560200 12372838

[B41] Studier F. W. (2005). Protein production by auto-induction in high density shaking cultures. Protein Expr. Purif. 41, 207–234. 10.1016/j.pep.2005.01.016 15915565

[B42] Teng T.-Y. (1990). Mounting of crystals for macromolecular crystallography in a free-standing thin film. J. Appl. Cryst. 23, 387–391. 10.1107/S0021889890005568

[B43] White H. Jencks W. P. (1976). Mechanism and specificity of succinyl-CoA:3-ketoacid coenzyme A transferase. J. Biol. Chem. 251, 1688–1699. 1254594

[B44] Whitty A. Fierke C. A. Jencks W. P. (1995). Role of binding energy with coenzyme A in catalysis by 3-oxoacid coenzyme A transferase. Biochemistry 34, 11678–11689. 10.1021/bi00037a005 7547900

[B45] Yang W. Drueckhammer D. G. (2003). Computational study of the citrate synthase catalyzed deprotonation of acetyl-coenzyme A and fluoroacetyl-coenzyme A: Demonstration of a layered quantum mechanical approach. J. Phys. Chem. B 107, 5986–5994. 10.1021/jp034717v

