# Supplementary material for: Functional Dissection of the Bipartite Active Site of the Class I Coenzyme A (CoA)-Transferase Succinyl-CoA:Acetate CoA-Transferase
Source: Front Chem. 2016 May 23;4:23. doi: 10.3389/fchem.2016.00023 (PMC4876117; doi:10.3389/fchem.2016.00023)
Supplement: Supplementary file 1 [file Presentation1.pdf]

Supplementary Material for: Functional Dissection of the Bipartite Active Site of the Class I Coenzyme A (CoA)-Transferase Succinyl-CoA:Acetate CoA-Transferase

Jesse R. Murphy, Elwood A. Mullins, and T. Joseph Kappock*

Department of Biochemistry, Purdue University, West Lafayette, Indiana 47907-2063, United States

E-mail: tjkappock@gmail.com

Phone: 765-494-8383. Fax: 765-494-1897

Running header

AarC Active Site

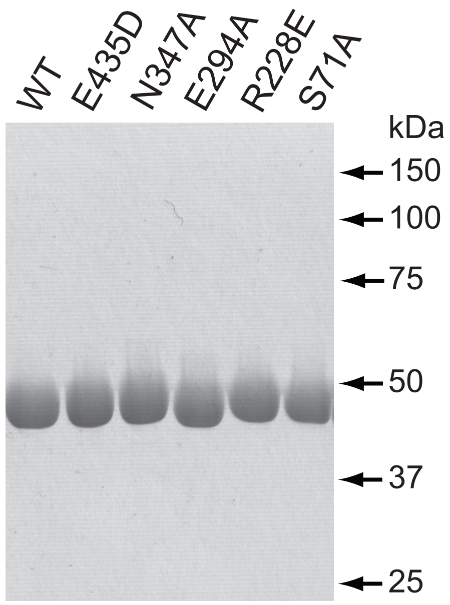

Figure S1: SDS-PAGE analysis of purified AarC and mutants. Each lane contains 5 μg of the wild-type (WT; AarC with C-terminal hexahistidine tag) or the indicated mutant protein isolated as described (Mullins and Kappock, 2012). Coomassie Blue stain was used to visualize protein bands. Size standard positions are indicated.

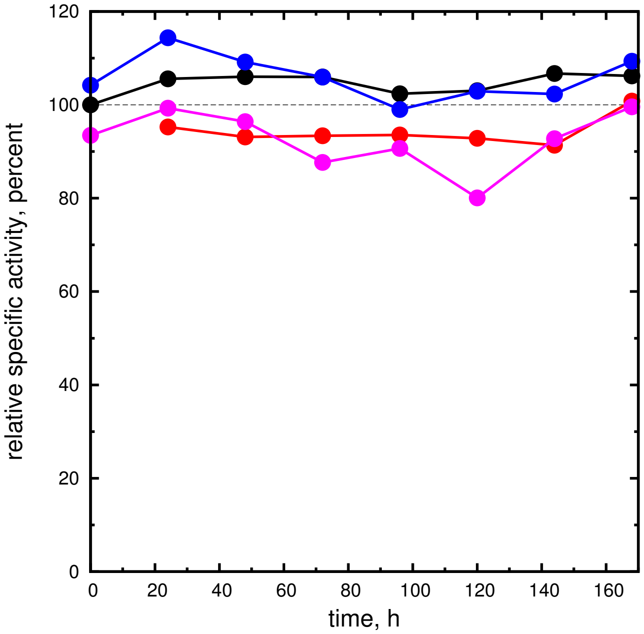

Figure S2: Attempted borohydride trapping of covalent adducts of AarC. Treatment with reductant is expected to inactivate AarC by irreversible reduction of either the Glu294 acetyl-glutamyl anhydride or glutamyl-CoA thioester adducts. Aliquots were withdrawn at the indicated times from either **1a** stability assays or no-**1a** controls and transferred to either 10 mM $NaBH_4$ or a water control. Specific activities are given relative to the zero-time point of a no- **1a** control that was not exposed to $NaBH_4$ (black symbols; 100% = 112 units/mg). Other experiments showed no time-dependent loss of SCACT activity: no **1a**, 10 mM $NaBH_4$ (red symbols; missing $t = 0$ point); 100 μM **1a**, no $NaBH_4$ (blue symbols), 100 μM **1a**, 10 mM $NaBH_4$ (purple symbols).

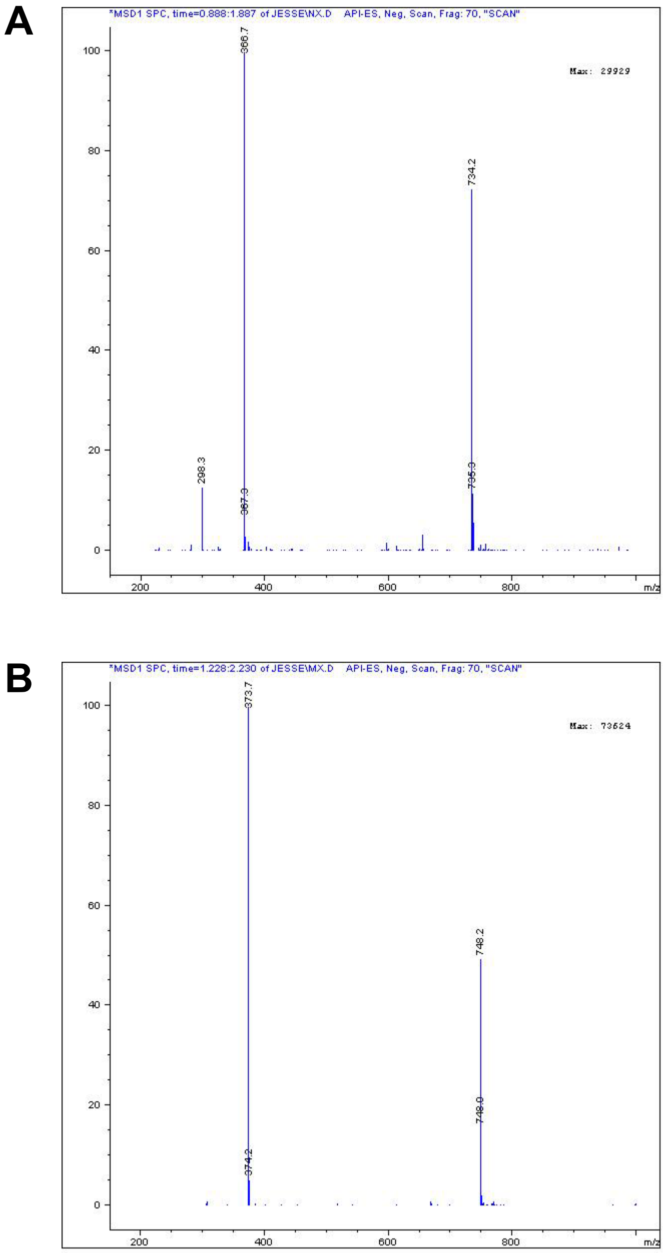

Figure S3: LCMS analysis (negative ion mode) of CoA analogues **2a** and **3a**. (**A**) Compound **2a** showed peaks at m/z 748 and 373.5, corresponding to singly and doubly deprotonated forms (expected parent molecule mass 749.16 Da), presumably one or more of the phosphate moieties. (**B**) Compound **3a** showed peaks at m/z 734 and 366.5, corresponding to singly and doubly deprotonated forms (expected parent molecule mass 735.14 Da).

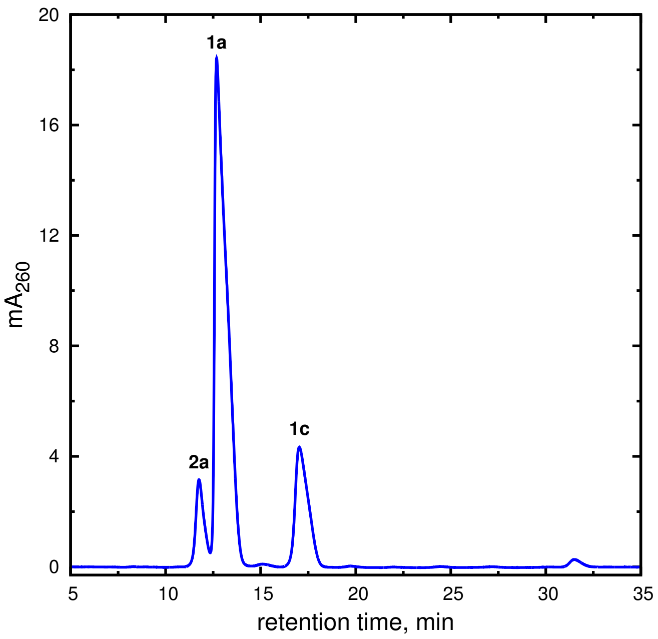

Figure S4: HPLC analysis of synthetic **1a** and **2a**. A 9:1 mixture of **1a** and **2a** was injected at $t = 0$ and the absorbance profile at 260 nm was recorded (mA = milli AU). Conditions were otherwise identical to those used to quantitate analogues in the **1a** stability assay (Figure 7). Peaks observed under these conditions were assigned using single-compound injections.

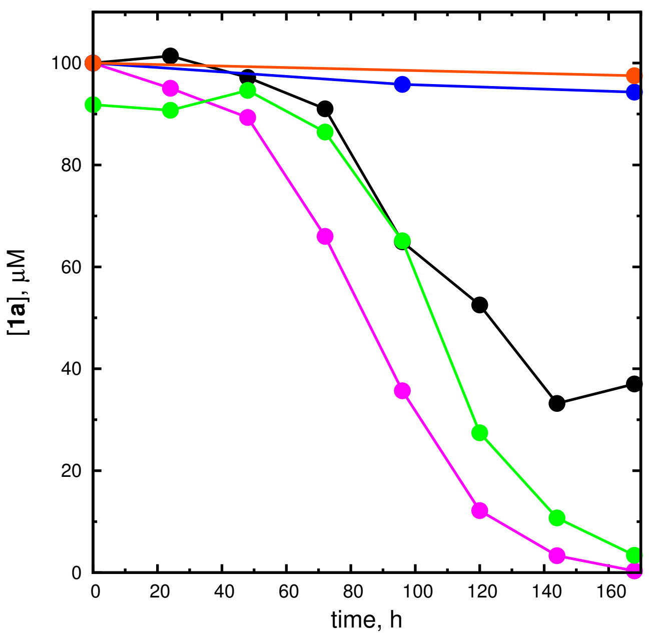

Figure S5: Active enzyme is not required for **1a** degradation. Absorbance spectra or HPLC analysis after heat quenching was used to monitor the decay of **1a** (initially 100 μM) in reaction mixtures containing: 10 μM AarC, magenta (HPLC) and green (A_{260}) symbols; 10 μM AarC-E294A, black symbols; no enzyme, blue symbols; and 10 μM AarC that was sterile-filtered immediately after mixing, red symbols.

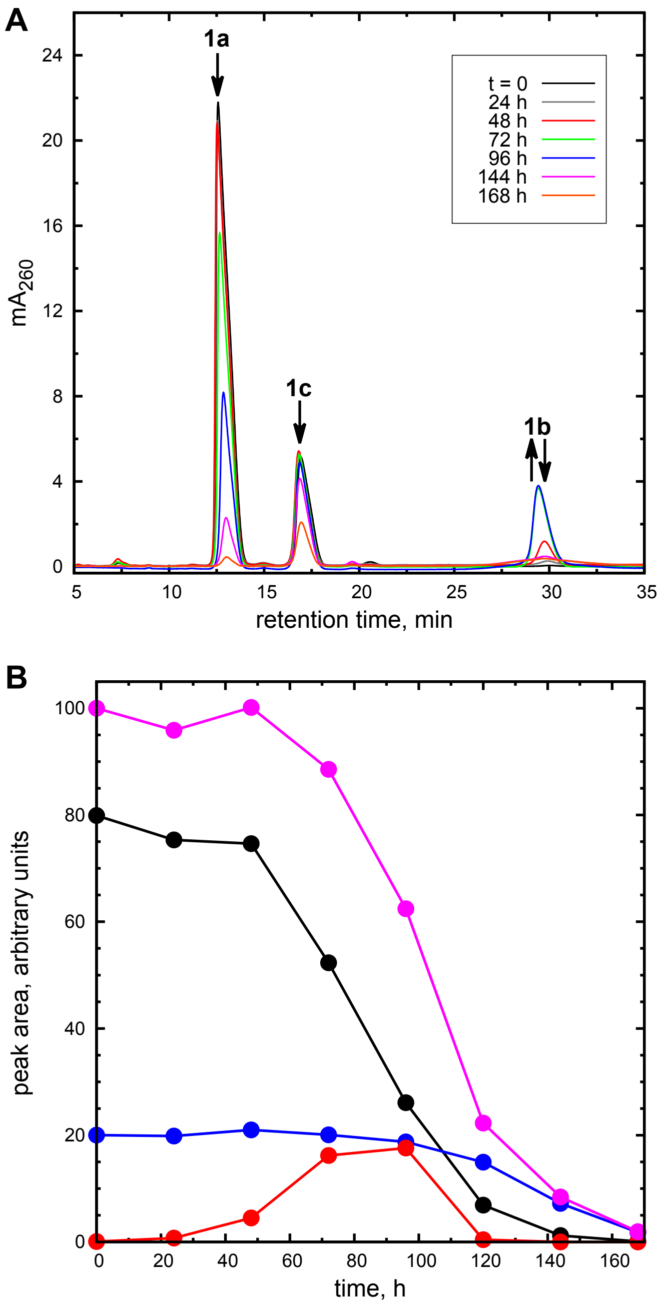

Figure S6: Transient formation of **1b**. (**A**) Sequence of HPLC chromatograms for a representative **1a** degradation time course. The peak at 29.4 min was found to contain a species consistent with **1b**, with m/z 712.17 (Figure S8); m/z 712.21 expected ($C_{24}H_{40}N_7O_{14}P_2^+$). Compound **2a** was not detected in this or other HPLC experiments. (**B**) Peak areas for the chromatograms shown in panel A (total $t = 0$ peak area set arbitrarily to 100): **1a**, black symbols; **1b**, red symbols; **1c**, blue symbols; total peak area, magenta. **1b** appears to be formed primarily from **1a** not **1c**.

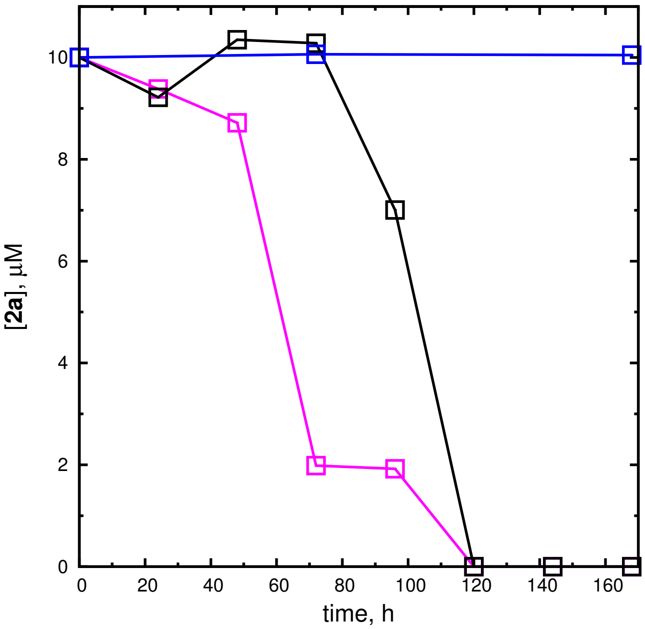

Figure S7: Compound **2a** decomposes in unfiltered reaction mixtures containing AarC. HPLC analysis after heat quenching was used to monitor the decay of **2a** (initially 10 μM) in reaction mixtures containing: 10 μM AarC, magenta symbols; 10 μM AarC-E294A, black symbols; no enzyme, blue symbols. Only the no-enzyme control contained detectable **2a** after 96 h (detection threshold ~1 μM).

Figure S8: MALDI-TOF detection of compounds formed during **1a** decomposition. Spectra acquired over 168 h showed an increasing proportion of species with smaller m/z values, many of which were not identified. Spectra (positive ion mode) obtained using complete reaction mixtures from $t = 0$ (blue spectrum) and 96 h (red spectrum) are superimposed. Boxes highlight **1a** peaks and circles new peaks consistent with the production of **2a** (expect m/z 750.17 for $[M{+}H]^+$, m/z 788.12 for $[M{+}K]^+$) or **1b** (expect m/z 719.21 for $[M{+}H]^+$, m/z 750.17 for $[M{+}K]^+$). Note that the **2a** and monopotassium **1b** ions are isobaric. The largest new peaks (m/z 712, 750, and 788) were only detected at 48, 72, and 96 h and appeared to correspond to the $[M{+}H]^+$, $[M{+}K]^+$, and $[M{+}2K]^+$ ions of **1b**. This pattern is consistent with transient production of **1b** as an intermediate in a multi-step **1a** degradation process.

Efforts to identify **1a**-derived compounds were hampered by a mass ambiguity: the replacement of a phosphoryl group with a hydrogen leads to a loss of 80 amu, as does the replacement of an acetyl group and a potassium with two hydrogens. As one consequence of this ambiguity, the all-protonated **2a** ion is isobaric with the monopotassium **1b** ion (m/z 750.17). Cleanup attempts employing cation exchange or solid phase extraction resulted in the loss of all previously detected ions (data not shown).

All MS spectra were searched for ions consistent with known or hypothetical compounds that might be produced during **1a** decomposition. The first search was for ions consistent with shortening of the aminopentanone moiety in **1a** or **1b**. Singly charged ions (containing 0, 1, or 2 potassium atoms) consistent with **3a**, **4a**, or dephosphorylated (Figure 2; **3b** and **4b**, respectively) products were not detected. Ions consistent with oxygen atom addition, with or without oxidation (+14 or +16 amu, respectively), to **3a**–**c** or **4a**–**c** were also not detected. The second search was for ions appearing transiently during **1a** decomposition. MALDI-TOF spectra acquired at 48, 72, and 96 h also contained new peaks at m/z 766 and 804, which were consistent with either (a) $[M{+}K]^+$ and $[M{+}2K]^+$ ions of **1b** plus an oxygen atom or (b) $[M{+}H]^+$ and $[M{+}K]^+$ ions of **2a** plus an oxygen atom. These species are consistent with compounds **5b** and **6a** (Figure 10), respectively, and are consistent with the working hypothesis discussed in the main text.

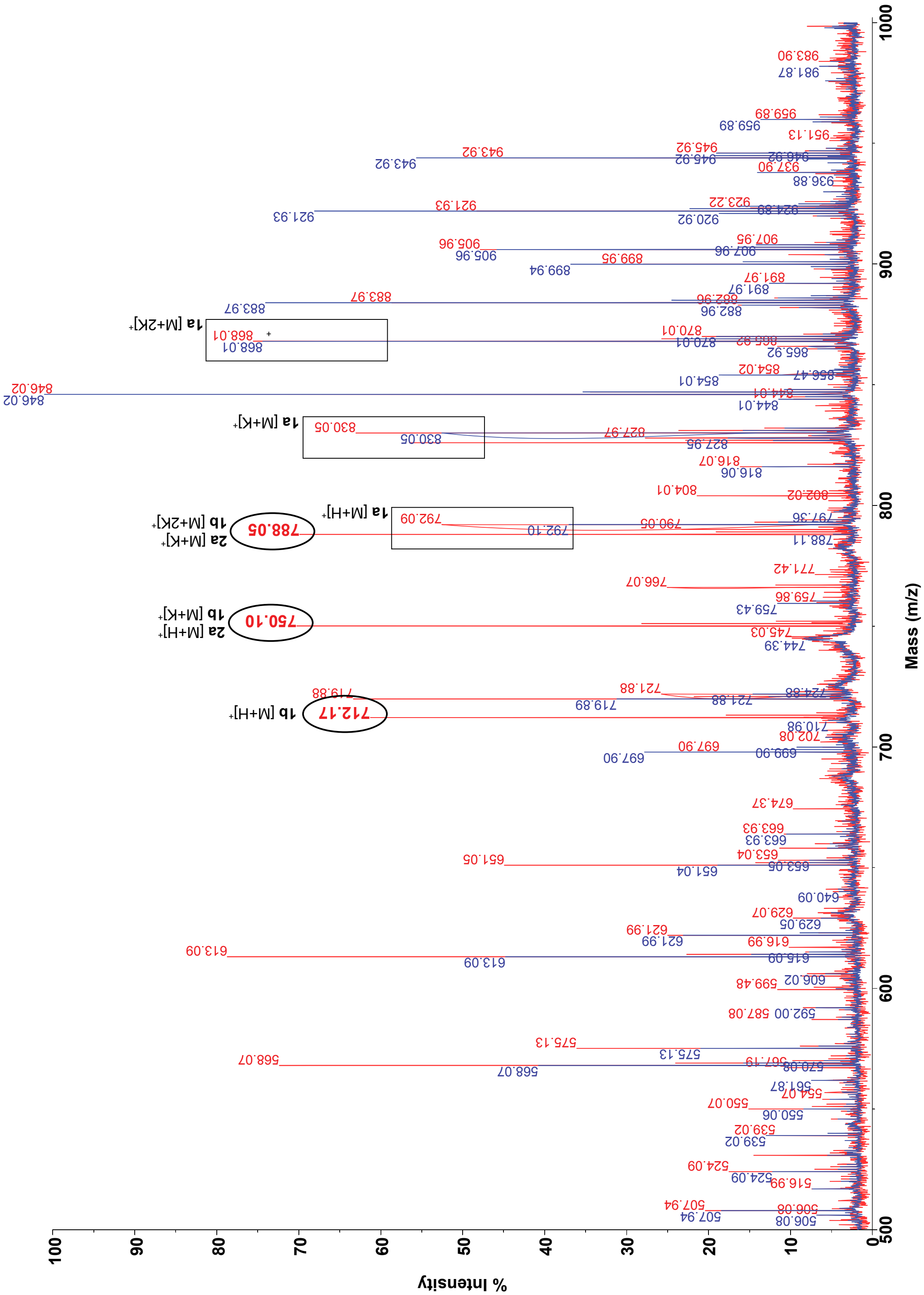
% Intensity
Mass (m/z)
1b [M+H]+
712.17
2a [M+H]+
1b [M+K]+
750.10
2a [M+K]+
1b [M+2K]+
788.05
1a [M+H]+
1a [M+K]+
1a [M+2K]+

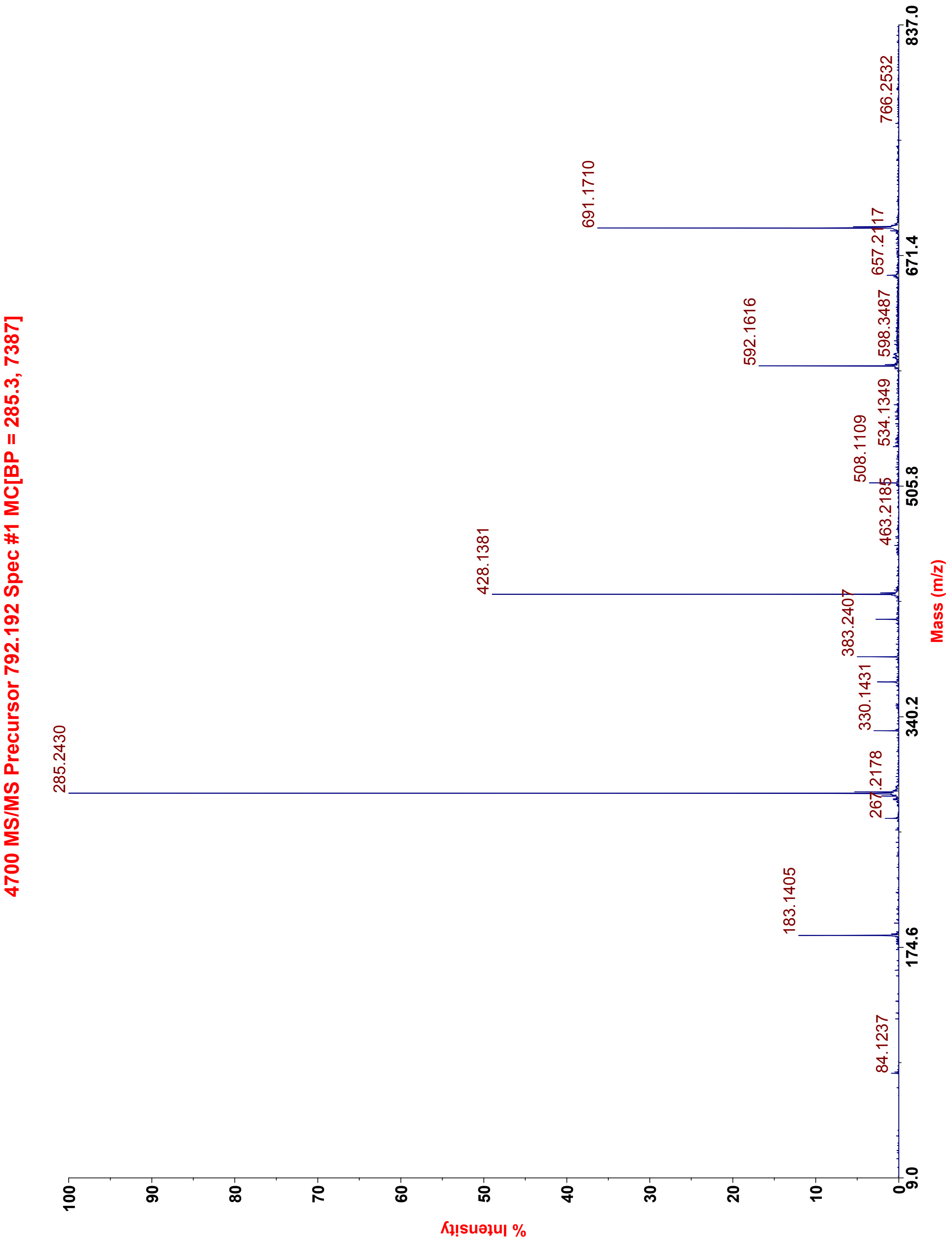

Figure S9: MS-MS spectrum of synthetic **1a**.

Figure S10: MALDI-MS/MS (positive ion mode) fragmentation of a m/z 750.17 ion obtained from synthetic **2a**. Potential daughter ion structures, chemical formulae, and computed m/z values are provided for prominent peaks. Protonation is presumed to occur at adenine N1 (pK_a ~3.6; Kapinos et al., 2011) in daughter ions that possess an adenine ring, but is speculative for others.

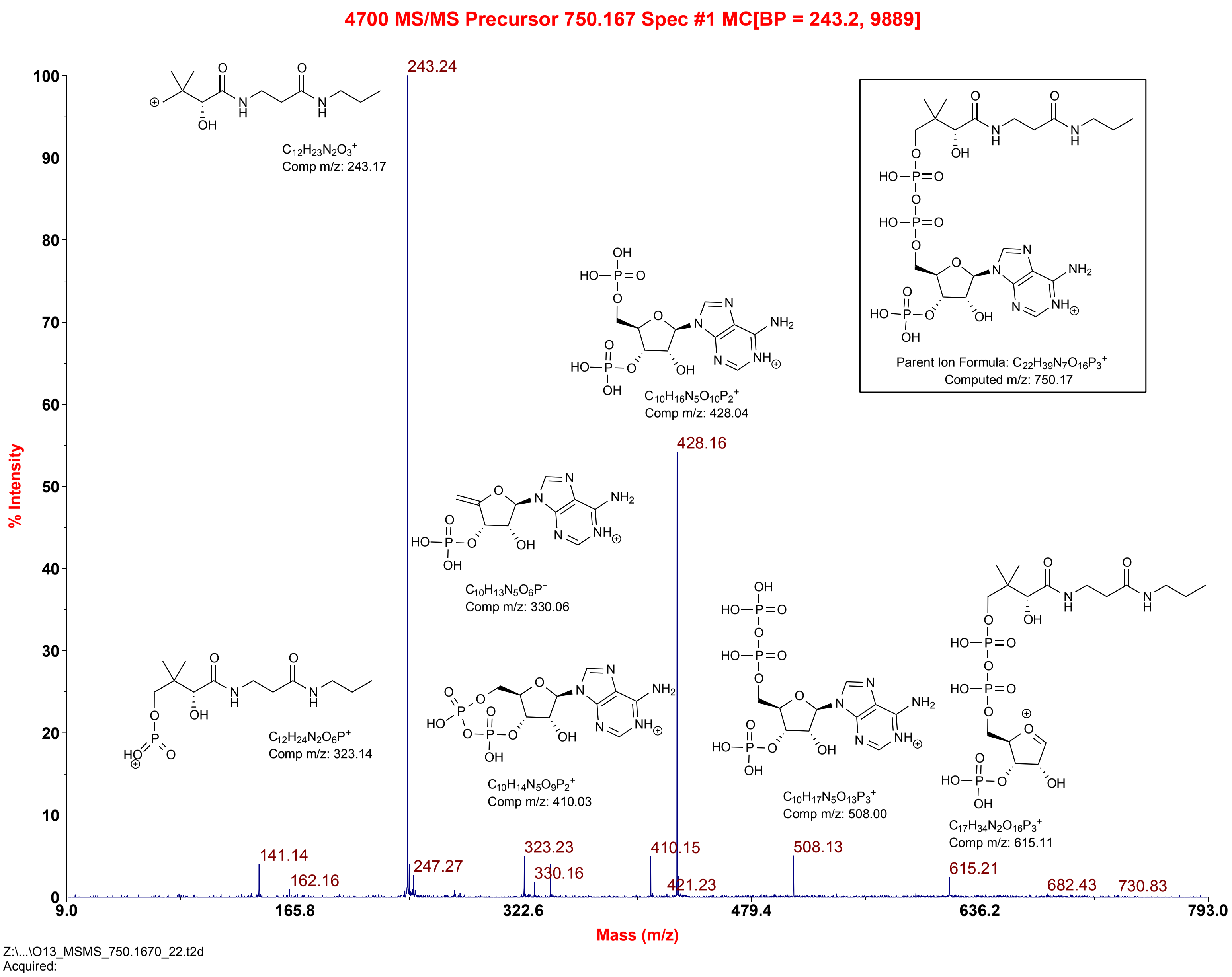
4700 MS/MS Precursor 750.167 Spec #1 MC[BP = 243.2, 9889]
% Intensity
Mass (m/z)
243.24
428.16
141.14
162.16
247.27
323.23
330.16
410.15
421.23
508.13
615.21
682.43
730.83
C12H23N2O3+
Comp m/z: 243.17
C10H16N5O10P2+
Comp m/z: 428.04
C10H13N5O6P+
Comp m/z: 330.06
C12H24N2O6P+
Comp m/z: 323.14
C10H14N5O9P2+
Comp m/z: 410.03
C10H17N5O13P3+
Comp m/z: 508.00
C17H34N2O16P3+
Comp m/z: 615.11
Parent Ion Formula: C22H39N7O16P3+
Computed m/z: 750.17
Z:\...\O13_MSMS_750.1670_22.t2d
Acquired:

Figure S11: MS/MS analysis of a candidate **1b** ion formed during **1a** decomposition. MALDI-TOF analysis (Figure S8) cannot discriminate between the isobaric **1b** $[M+K]^+$ and **2a** $[M+H]^+$ ions (m/z 750.17). MS/MS daughter ion fragmentation patterns were therefore recorded and compared to those for authentic **1a** (Figure S9) and **2a** (Figure S10). The MS/MS spectrum of the 750.17 ion was obtained from the 96 h sample (red spectrum, Figure S8). The observed MS/MS spectrum is a poor match to the MS/MS spectrum of synthetic **2a** (Figure S10). Speculative structures for selected potassium **1b** daughter ions are given. Several daughter ions were observed unique to **1b** but none unique to **2a**, together with daughter ions that could derive from either compound. In addition, several prominent daughter ions in the synthetic **2a** spectrum (Figure S10) were not detected. While the possibility of a small amount of **2a** formation cannot be ruled out, **1b** is the best assignment of the m/z 750.17 ion.

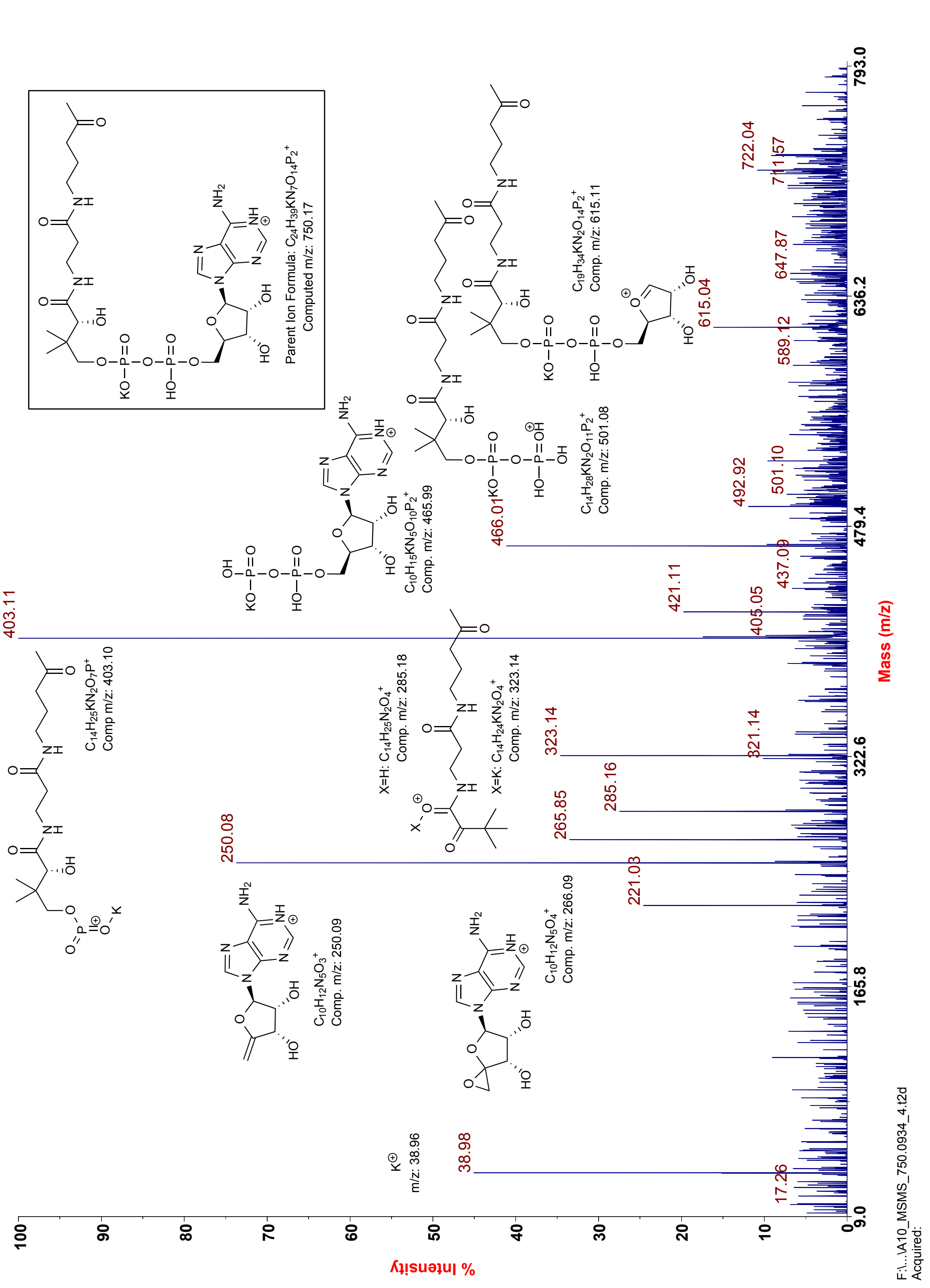
% Intensity
Mass (m/z)
K⊕ m/z: 38.96
38.98
17.26
221.03
250.08
265.85
285.16
321.14
323.14
403.11
405.05
421.11
437.09
466.01
492.92
501.10
589.12
615.04
647.87
711.57
722.04
9.0
165.8
322.6
479.4
636.2
793.0
$C_{14}H_{25}KN_2O_7P^+$ Comp m/z: 403.10
$C_{10}H_{12}N_5O_3^+$ Comp. m/z: 250.09
$C_{10}H_{12}N_5O_4^+$ Comp. m/z: 266.09
X=H: $C_{14}H_{25}N_2O_4^+$ Comp. m/z: 285.18
X=K: $C_{14}H_{24}KN_2O_4^+$ Comp. m/z: 323.14
$C_{10}H_{15}KN_5O_{10}P_2^+$ Comp. m/z: 465.99
$C_{14}H_{28}KN_2O_{11}P_2^+$ Comp. m/z: 501.08
$C_{19}H_{34}KN_2O_{14}P_2^+$ Comp. m/z: 615.11
Parent Ion Formula: $C_{24}H_{39}KN_7O_{14}P_2^+$
Computed m/z: 750.17
F:\...\A10_MSMS_750.0934_4.t2d
Acquired:

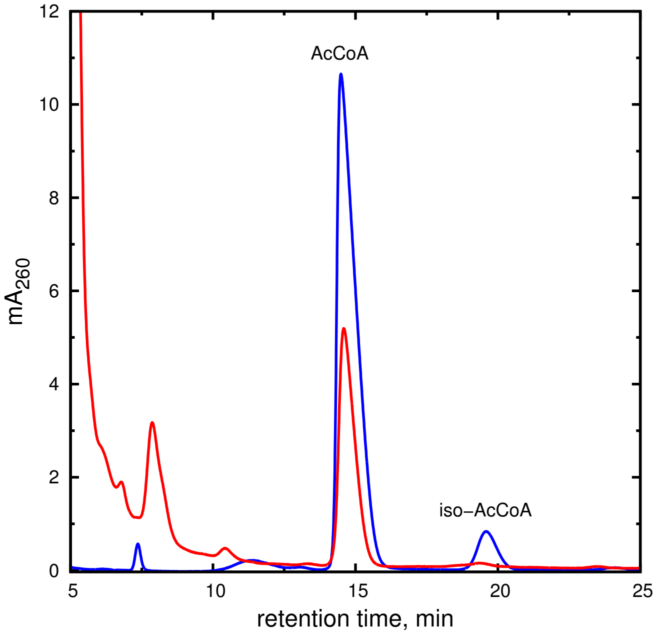

Figure S12: Acetate is produced during **1a** decomposition. ACS was used to convert acetate present in **1a** stability assay reaction mixtures to AcCoA, which was identified by HPLC analysis. Detection of AcCoA confirms that acetate is a product of **1a** decomposition. Blue trace, chromatogram obtained using an authentic AcCoA standard. Red trace, chromatogram demonstrating ACS-mediated formation of AcCoA from **1a** stability assays (96 h time point).

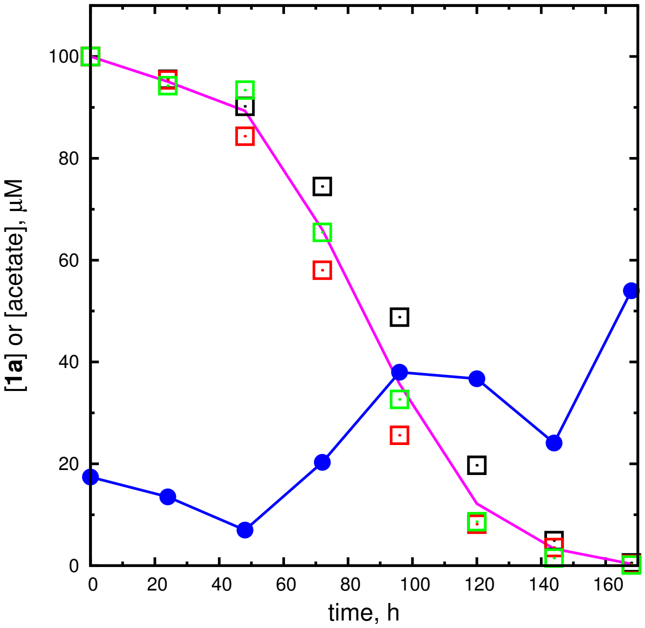

Figure S13: Alternative method for quantitation of acetate produced from **1a**. Intact **1a** was detected and quantitated by HPLC in three independent time courses (open squares of different colors). The average [**1a**] is depicted here with a magenta line, and in Figure 7 with a line and error bars. The blue filled circles depict acetate detected using ACS in aliquots from **1a** stability assay #3 (green open squares).

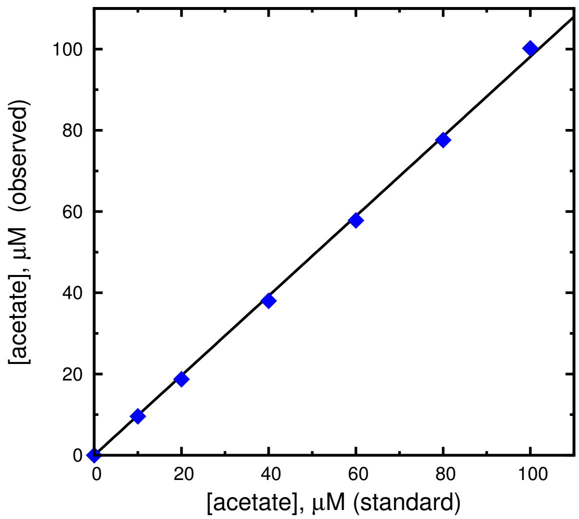

Figure S14: Linear response of acetate determination assay. A standard acetate solution was analyzed using the AK-dependent PK/LDH ATPase assay. A final volume of 70 μL contained $0 - 100$ μM acetate ($0 - 7$ nmol acetate total). The ΔA_{340} value recorded in the zero-acetate sample was used as a background for the other concentrations (symbols). A linear fit to the data, forced to pass through the origin, has slope 0.981 ± 0.009 (solid line).

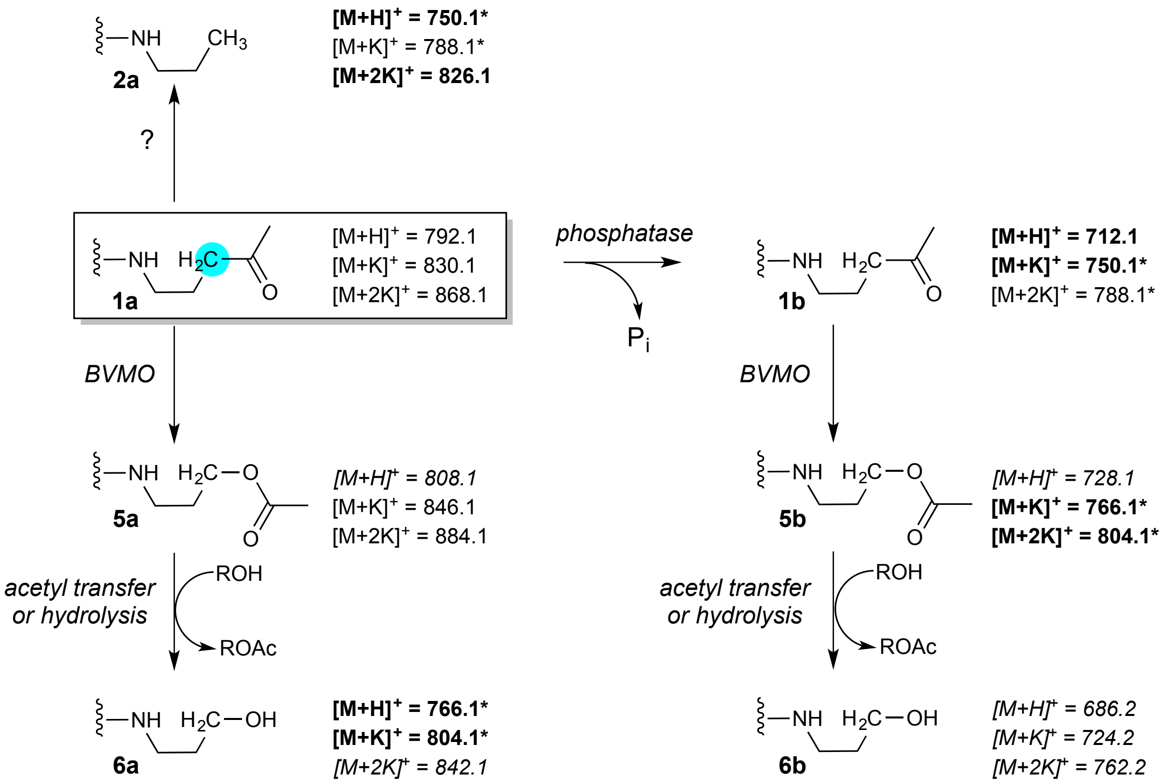

Figure S15: Speculative pathways for **1a** degradation by microbes. If BVMO is present, it could act on either **1a** or **1b**. Plain m/z values, ions detected at time points including $t = 0$. Bold m/z values, ions detected at time points other than $t = 0$. Italicized m/z values, ions not detected. Asterices denote m/z values that are consistent with multiple ions.
